# Abnormal White Matter Microstructure in the Limbic System Is Associated With Tuberous Sclerosis Complex-Associated Neuropsychiatric Disorders

**DOI:** 10.3389/fneur.2022.782479

**Published:** 2022-03-14

**Authors:** Akemi Sato, Koji Tominaga, Yoshiko Iwatani, Yoko Kato, Mari Wataya-Kaneda, Kai Makita, Kiyotaka Nemoto, Masako Taniike, Kuriko Kagitani-Shimono

**Affiliations:** ^1^United Graduate School of Child Development, Osaka University, Osaka, Japan; ^2^Molecular Research Center for Children's Mental Development, Osaka University Graduate School of Medicine, Osaka, Japan; ^3^Department of Pediatrics, Osaka University Graduate School of Medicine, Osaka, Japan; ^4^Division of Health Science, Department of Neurocutaneous Medicine, Graduate School of Medicine, Osaka University, Osaka, Japan; ^5^Department of Dermatology, Graduate School of Medicine, Osaka University, Osaka, Japan; ^6^Research Center for Child Mental Development, University of Fukui, Fukui, Japan; ^7^Division of Clinical Medicine, Department of Psychiatry, Faculty of Medicine, University of Tsukuba, Tsukuba, Japan

**Keywords:** TAND, limbic system, white matter, MRI, DTI, TSC

## Abstract

**Objective:**

Tuberous sclerosis complex (TSC) is a genetic disease that arises from *TSC1* or *TSC2* abnormalities and induces the overactivation of the mammalian/mechanistic target of rapamycin pathways. The neurological symptoms of TSC include epilepsy and tuberous sclerosis complex-associated neuropsychiatric disorders (TAND). Although TAND affects TSC patients' quality of life, the specific region in the brain associated with TAND remains unknown. We examined the association between white matter microstructural abnormalities and TAND, using diffusion tensor imaging (DTI).

**Methods:**

A total of 19 subjects with TSC and 24 age-matched control subjects were enrolled. Tract-based spatial statistics (TBSS) were performed to assess group differences in fractional anisotropy (FA) between the TSC and control groups. Atlas-based association analysis was performed to reveal TAND-related white matter in subjects with TSC. Multiple linear regression was performed to evaluate the association between TAND and the DTI parameters; FA and mean diffusivity in seven target regions and projection fibers.

**Results:**

The TBSS showed significantly reduced FA in the right hemisphere and particularly in the inferior frontal occipital fasciculus (IFOF), inferior longitudinal fasciculus (ILF), superior longitudinal fasciculus (SLF), uncinate fasciculus (UF), and genu of corpus callosum (CC) in the TSC group relative to the control group. In the association analysis, intellectual disability was widely associated with all target regions. In contrast, behavioral problems and autistic features were associated with the limbic system white matter and anterior limb of the internal capsule (ALIC) and CC.

**Conclusion:**

The disruption of white matter integrity may induce underconnectivity between cortical and subcortical regions. These findings suggest that TANDs are not the result of an abnormality in a specific brain region, but rather caused by connectivity dysfunction as a network disorder. This study indicates that abnormal white matter connectivity including the limbic system is relevant to TAND. The analysis of brain and behavior relationship is a feasible approach to reveal TAND related white matter and neural networks. TAND should be carefully assessed and treated at an early stage.

## Introduction

Tuberous sclerosis complex (TSC) is an autosomal dominant disorder characterized by multiple organ hamartomas, including the brain, heart, skin, kidney, and liver (1). *TSC1* and *TSC2* encode hamartin and tuberin, respectively (1). The hamartin-tuberin complex inhibits the mammalian/mechanistic target of rapamycin (mTOR) pathways, which play a role in controlling cellular growth, cell proliferation, and suppressing tumor formation (2). Overactivation and dysregulation of the mTOR pathway, which are caused by *TSC1* or *TSC2* abnormalities, promote tumor formation. The variety of tumors that occur in TSCs are a result of this cellular growth dysregulation (3). The neurological manifestations of TSC include cortical tubers noted in more than 80% (1, 4), subependymal nodules (SEN) noted in 70–84% (5), subependymal giant cell astrocytomas (SEGAs) noted in 5–15% (4), and epilepsy noted in 80–90% of patients (6, 7). Tuberous sclerosis complex associated neuropsychiatric disorders (TAND) is a new term coined at the 2012 International TSC Consensus Conference to unite the neurological manifestations of TSC, such as intellectual disability (ID), autistic spectrum disorders (ASD), behavioral difficulties (aggression, anxiety, and self-injury), and other psychiatric symptoms (8, 9). Although TAND highly affects the individual with TSC and their quality of life during their lifetime (10), the neuropsychiatric manifestations remain highly under-identified and under-treated (8, 9, 11). Approximately 20–50% of individuals with TSC have ASD (9–12) and epilepsy has previously been considered a causal factor for the subsequent development of TAND, including ASD and ID (12). Evidence suggests that early-onset epilepsy and a history of infantile spasms (ISs) are the risk factors for developmental delay and intellectual disability in TSC (13, 14), whether these play a significant role in the development of ASD as well is not clear (14). The neurological pathophysiology in TSC, such as that relating to epilepsy and TAND, remains unknown (3).

Diffusion tensor imaging (DTI) is an MRI imaging technique that provides information about white matter microstructural integrity by examining the average water diffusion, at the tissue microscopic level (15–17). Two main DTI measures are commonly used: fractional anisotropy (FA) and the mean diffusivity (MD). FA is the degree of anisotropy and is extracted from the eigenvalues of the diffusion tensors, λ_1_, λ_2_, and λ_3_. Reduced FA levels reflect decreased white matter integrity (18). MD is the average of the eigenvalues of the three diffusions, as follows: $\frac{\left(\lambda 1+\lambda 2+\lambda 3\right)}{3}$. When neurons or myelin sheaths are damaged, FA decreases and MD increases. A DTI study revealed significantly reduced FA in the corpus callosum in TSC subjects relative to controls (19). However, TAND- related white matter tracts remain unknown (20). The human limbic system is involved in behavior, emotion, and memory (21). The limbic system white matter consists of three major tracts: the cingulum, the stria terminalis, and the fornix (21). In addition, the uncinate fasciculus, which connects the orbitofrontal cortex (OFC) to the temporal lobe, is a crucial white matter associated with social-emotional-cognitive function and several psychiatric disorders (22–24). The Papez Circuit is relevant to emotion and memory, which consists of the following paths; hippocampal formation—fornix—mamillary body—anterior thalamic nucleus—thalamocingulate tract: cingulate gyrus cingulum—parahippocampal gyrus, and return to hippocampal formation (22, 25). The anterior limb of the internal capsule (ALIC) receives prefrontal cortex fiber projections to subcortical regions, and is involved in thalamocingulate tract (25, 26). Recently, the association with specific neural network and neuropsychiatric disorders has been reported (26). Therefore, to examine the white matter connectivity of the limbic system and projection fibers in subjects with TSC is critical for understanding the mechanism of TAND.

This study aimed to investigate the differences in the white matter based on the severity and variety of TAND in subjects with TSC. In addition to autism features, intellectual disability, relationship difficulties, and behavioral problems are included in the umbrella term of TAND (8), as a qualitative study reported difficulties with relationships and a lack of empathy in young adult patients with TSC (27). We aimed to reveal the association between autistic features or maladaptive behavior and white matter microstructural abnormalities, particularly in the limbic system and several major white matter in subjects with TSC. We hypothesized that decreased white matter integrity in the limbic system could be associated with the parts of TAND manifestations in subjects with TSC.

## Methods

### Study Design

This study was conducted with a prospective and cross-sectional design. To estimate the microstructural changes in the developing brain, we had a target age rating from 6 to 28 years. Participants with TSC were recruited from the Department of Pediatrics or Dermatology at Osaka University Hospital (Osaka, Japan) between September 2019 and January 2021.

### Subjects

All subjects met the diagnostic criteria for definite TSC. Subjects with a history of brain surgery (either epilepsy surgery or resection of subependymal giant cell astrocytomas) were excluded. Clinical information about epilepsy was obtained retrospectively from the medical records, including the following: history of ISs, epilepsy onset in months or the severity of epilepsy, the number of current anti- epileptic drugs (AEDs), and the use of mTOR inhibitors. Epilepsy type was classified using ILAE classification (28) as follows: developmental and epileptic encephalopathies (DEE) and focal epilepsy (FE), and the severity was classified as follows: 0, never; 1, well controlled (seizure-free for more than a year); and 2, refractory epilepsy. The cortical tubers were identified in fluid-attenuated inversion recovery (FLAIR) images. In addition, subjects with typical development who had no history of neurological conditions, including epilepsy or ASD, were recruited as control volunteers from neighboring cities. All participants and/or their guardians provided written informed consent to participate in the study. This study was approved by the Institutional Review Board of Osaka University Hospital (No. 18491) and was conducted according to the principles of the Declaration of Helsinki. All subjects were informed of the study and provided consent before study entry.

### Cognitive and Behavioral Assessments

#### ASD Symptoms

Diagnoses of ASD were confirmed using the criteria of the Diagnostic and Statistical Manual of Mental Disorders, Fifth Edition (DSM-5; APA), by two experienced pediatric neurologists in subjects with TSC. We used the Japanese version of the Social Responsiveness Scale-2 (SRS-2) (29) in patients aged 6–18 years old to confirm ASD diagnosis (SRST score ≥ 80). Uematsu et al. (30) confirmed that SRS-2 is a useful tool to assess autism-related behavior in children with TSC. The SRS-2, which was reported by parents, consists of three domains: weakness of socialization, weakness of communication, and repetitive behaviors (31). Higher SRS-T scores indicate more ASD-like behaviors. Because the Japanese version of SRS-2 is not available for patients aged > 18 years, we used the Parent-interview ASD Rating Scale-Text Revision (PARS-TR) (32) in subjects aged > 18 years and confirmed the exceeding cut-off value. In addition, the Japanese version of the Autism-spectrum Quotient (AQ) (33) was performed by parents of control subjects to screen for ASD.

#### Intellectual Ability

Full-scale intelligence quotients (FSIQ) were measured with the age-appropriate Wechsler Intelligence scale for both TSC and control subjects (WISC-IV, WAIS-IV; the Japanese versions were used). For subjects with TSC who could not complete a Wechsler Intelligence scale due to severe intellectual disability, estimated IQ was calculated using the Japanese version (34) of the Vineland Adaptive Behavior Scales (VABS)-Second Edition (35). An estimated IQ of <70 was defined as an intellectual disability.

#### Vineland Adaptive Behavior Scale

VABS-Second Edition is a standardized questionnaire to measure adaptive behavior through semi-structured interviews. And it is used to support the diagnosis of ID, ASD, and developmental delay (34). The VABS consists of three different domains in adaptive behavior evaluation: communication, socialization, and daily living skills. Parents or caregivers of subjects with TSC answered these three domains about individuals with TSC. The socialization domain reflects the subject's interpersonal, play, leisure, and coping skills (36). A study reported that individuals with ASD have a significantly low score in the socialization domain, whereas individuals with ID have relatively flat profiles across all the three domains (37). In addition to adaptive behavior scales, we used the Maladaptive Behavior Index as an option to assess behavioral difficulties in subjects with TSC. This index consists of a total of 50 questions about internalizing problems (e.g., sleep difficulty, lack of eye contact, anxiety, and nervous), externalizing problems (e.g., hyperactivity, impulsivity, and aggression), and other problems (e.g., self-injury and sensory sensitivity).

#### TAND Check List

The parents or caregivers answered the Japanese version of the TAND checklist [CHECKLISTS - TANDem (tandconsortium.org)] (38). The TAND checklist was developed for clinical teams and families to support annual screening (8). The TAND check list consists of six levels: behavioral, psychiatric, intellectual, academic, neuropsychological, and psychosocial (8). The Vineland Maladaptive Behavior Index includes all behavior level questions (19 questions) of the TAND check list. Because the TAND checklist is not standardized, we used the standardized Maladaptive Behavior Index to represent behavior level difficulties in TAND.

To assesses the TAND manifestations we used the following scales: (1) estimated IQ, to assess intellectual disability (ID); (2) Vineland Maladaptive Behavior Index, to assess behavioral level difficulties; (3) SRS-T score to assess ASD feature; and (4) Vineland Socialization score to assess relationship difficulties.

### MRI Acquisition

MRI images were acquired using a 3.0 T MRI scanner (SIGNA Architect; GE Healthcare, Milwaukee, WI, USA) for all subjects (*n* = 43). Single-shot, spin-echo, echo-planar imaging (EPI) was performed. Axial diffusion-weighted images were acquired with the following parameters: echo time (TE) = 75 ms, repetition time (TR) = 6,000 ms, matrix size = 128 × 128, field of view (FOV) = 260 × 260 mm^2^, slice thickness = 3 mm, and number of slices = 50. Diffusion sensitization gradient was applied with 25 non-collinear gradient directions and a b value of 1,000 s/mm^2^, in addition to one non-diffusion-weighted scan. The children who could not keep still for the scanning were sedated with either pentobarbital, thiopental, pentazocine, levomepromazine, risperidone, triclofos, diazepam, midazolam, or a combination of the afore mentioned sedatives.

### DTI Processing

#### Pre-processing

All MRI processing and DTI analyses were performed using Functional MRI of the Brain (FMRIB) Software Library FSL6.0.4 (https://fsl.fmrib.ox.ac.uk/) (39). First, the raw DTI data were corrected for motion and eddy current effects using *eddy_correct*. Next, automatic brain extraction was performed using the brain extraction tool (BET), and a brain mask was created from the b0 image. FA/MD images were created using DTIFIT and checked for the presence of artifacts.

#### Extraction of DTI Parameters (FA/MD) for Analysis

FA images of each subject were aligned into a Montreal Neurological Institute (MNI) space, first with linear registration in the FMRIB's linear image registration tool (FLIRT), followed by a non-linear image registration tool (FNIRT). The dimension of the normalized FA image was 1 × 1 × 1 mm^3^. The mean FA value of each region was calculated using the FMRIB's *fslstats* tool. A total of 48 regions were labeled according to the Johns Hopkins University (JHU) ICBM-DTI-81 white matter atlas (40).

To extract mean MD values, we used non-linear registration parameters for FA images. The ICBM-DTI-81 white matter atlas was inversely transformed to each subject space using the FMRIB's *invwarp* and *applywarp* tools. Then, mean MD values were calculated based on subject-based atlas using *fslstats*.

#### Tract-Based Spatial Statistics

To evaluate group differences in FA values between the TSC (*n* = 19) and control (*n* = 24) groups, voxel-wise statistical analysis was performed using TBSS. High reproducibility and objectivity are advantages of the TBSS (41). By using the non-linear registration tool, all the FA images were aligned into a common space. Since this study consisted of subjects with a wide range of ages from 6 to 28 years, the common template used in FSL was not adapted. Hence, we chose the “most representative” option and all subject's FA images were registered to one another to find the most “typical” subject to be used as a target image to be aligned with all others (41). Next, the target image was transformed into the MNI152 space. To generate the mean FA images, all aligned FA images were averaged. To generate the mean FA skeleton, each mean FA image was thinned. The threshold of the mean FA skeleton was set to 0.2, and the FA images of each subject were projected onto this skeleton.

We used two types of atlases: the JHU ICBM-DTI-81 white matter atlas for the extraction of DTI parameters, and the JHU white Matter Tractography Atlas for TBSS. Hence, the sagittal stratum (SS) in ICBM-DTI-81 is the same region as the inferior longitudinal fasciculus (ILF) and inferior frontal occipital fasciculus (IFOF) in the JHU White Matter Tractography Atlas (40).

### Statistical Analysis

The Mann-Whitney U test, chi-squared (χ^2^) test was used to compare the group descriptive data of the groups. Using *randomize*, which is an FSL tool for non-parametric permutation inference, voxel-wise statistical analysis was performed. Voxels were extracted where the FA value was significantly different between skeletons, using a general linear model (GLM) (41). Five-thousand permutations were applied, and the threshold was set to *p* < 0.05. The results were corrected for multiple comparisons using threshold-free cluster enhancement (TFCE) with family wise error (FWE) rate controlled with a cluster size of voxel > 10. Age and sex were used as covariates in the design matrix. Using the JHU White Matter Tractography Atlas, the anatomical regions of significant clusters were identified.

We hypothesized that the limbic area white matter would be associated with TAND, and we targeted seven regions based on our hypothesis: (1) the body of the fornix (FNX), (2) sagittal stratum (SS), (3) stria terminalis (ST), (4) cingulum in the cingulate gyrus (CGC), (5) superior longitudinal fasciculus (SLF), (6) uncinate fasciculus (UF), and (7) splenium of the corpus callosum (CC).

Correlations between DTI parameters (FA/MD) and behavior assessments (estimated IQ/socialization score /maladaptive behavior index/SRS-T score) were determined using Spearman's correlation coefficient (rs). Significance was established at *p* < 0.05. To reveal TAND related white matter in the TSC group (*n* = 19), multiple linear regression was performed to evaluate the association between independent variables (behavior) and dependent variables (FA/MD); age and sex were adjusted as covariates.

The model was as follows:


$$\begin{array}{l} \text{y}\;=\;\beta_{0}\;+\;\beta_{1}\text{Behavior }+\;\beta_{2}\text{a}g\text{e}\;+\;\beta_{3}\text{s}e\text{x} \end{array}$$

where y = FA/MD value, β_**1**_, β_**2**_, β_**3**_= coefficient, β_**0**_=intercept, *Behavior* = estimated IQ/socialization score/maladaptive behavior index/SRS-T score. In addition to above-mentioned 7 target regions, we examined the association between behavior and the anterior limb of internal capsule (ALIC), which connects prefrontal cortex to subcortical structures. To reveal active epilepsy influence on DTI parameters (FA/MD), we added refractory epilepsy presence (yes = 1, no = 0) as an independent variable in the above model. Age and sex were adjusted as covariates. All statistical analyses were performed using STATA/IC16.1 (Stata Corp LLC, College Station, Texas, USA).

## Results

### Clinical Risk for TAND Manifestations

**Figure 1:**
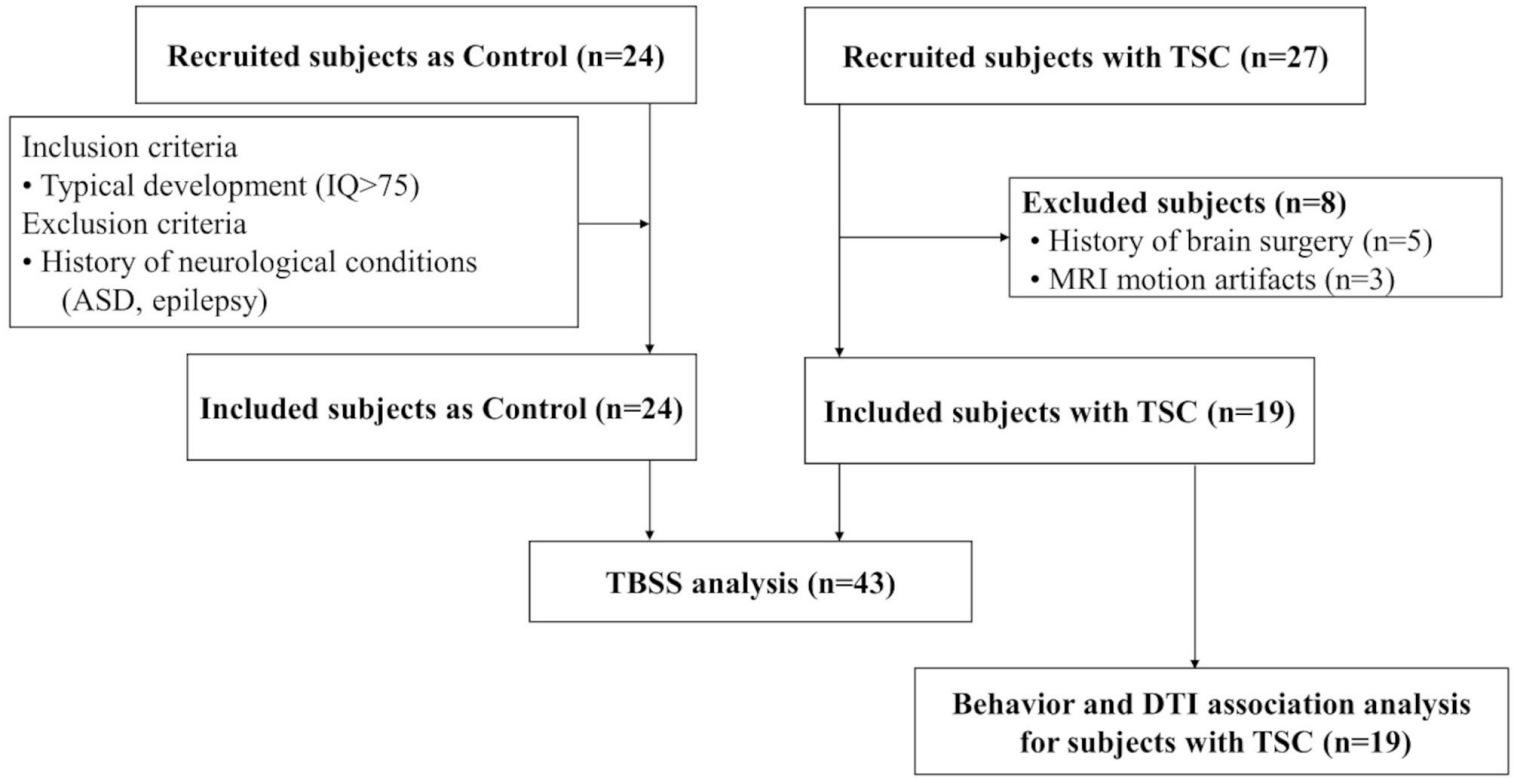
A total of 19 subjects with TSC and 24 age-matched controls are enrolled. To compare the white matter microstructural integrity between the TSC and control groups, tract-based spatial statistics (TBSS) are performed. Next, to reveal TAND-related white matter, association analysis is performed between behavior assessments and DTI parameters (FA/MD) for subjects with TSC (*n* = 19).

We included 43 subjects in our analysis. A total of 19 subjects with TSC (mean age ± SD; 12 ± 6 years, range 6–28) were enrolled. Of the 27 subjects with TSC recruited to the TSC group, 8 were excluded (5 due to a history of brain surgery and, 3 due to MRI motion artifacts), leaving a final sample of 19 subjects in the TSC group. In addition, 24 age-matched control subjects (mean age ± SD; 12 ± 5 years, range 6–27) were recruited, and none were excluded (Figure 1). The TSC and control groups did not differ significantly in age or sex, but the estimated IQ was significantly lower in the TSC group (z = 4.4, *p* < 0.001; Table 1). The main clinical features of the 19 subjects with TSC are summarized in Table 2. Fourteen subjects (74%) had concomitant epilepsy, 6 and 11 of whom had DEE and FE, respectively. The seizure severity was classified to well controlled (seizure-free for more than a year) epilepsy in 9 subjects, and refractory epilepsy in 5 subjects. Refractory epilepsy frequency ranged from more than once a week to less than once per month. Among those with epilepsy, 6 (43%) had a history of ISs. All 19 subjects with TSC had cortical tubers on FLAIR images, 17 with multiple cortical tubers in both hemispheres and 2 with multiple cortical tubers in left hemisphere. FSIQ was unavailable for four subjects with TSC, because their intellectual functioning was below the floor of the Wechsler scales, although we estimated their IQ by using the Vineland Adaptive Behavior Score. Eight subjects (42%) had comorbid ASD of which 6 (aged 6–15 years) had an SRS-T score over 80, and two adult subjects (23 and 28 years old) exceeded the PARS-TR cut-off value. Chi-squared (χ^2^) test indicated that early- onset epilepsy (before 2 years) and a history of ISs were risk factors for ID (estimated IQ <70; *p* = 0.04 and, *p* = 0.03, respectively), but were not risk factors for ASD features (SRS-T score over 80; *p* = 0.12 and, *p* = 0.17, respectively). In addition, there was a positive correlation between the Maladaptive Behavior Index and the SRS-T score (rs = 0.76, *p* < 0.001). The type and rate of TAND behavioral level difficulties in subjects with TSC are summarized in Supplementary Material 1. Five subjects (26%) were treated with mTOR inhibitors at the time of MRI in the present study. The Mann-Whitney U test indicated that there was no significant difference between the TSC patients receiving mTOR inhibitors (*n* = 5) and those not receiving mTOR inhibitors (*n* = 14) in the maladaptive behavior index score (with mTOR inhibitor, mean ± SD: 19 ± 1.5; without mTOR inhibitor: 18 ± 2.1; z = −1.02, *p* = 0.31), SRS-T score (with mTOR inhibitor, mean ± SD: 73 ± 8.8; without mTOR inhibitor: 68 ± 19, z = −0.57, *p* = 0.57), estimated IQ (with mTOR inhibitor, mean ± SD: 56 ± 31; without mTOR inhibitor: 73 ± 32; z = 0.97, *p* = 0.33).

**Table 1:** Descriptive data of the TSC and control groups.

	**TSC (*****n*** **=** **19)**		**Control (*****n*** **=** **24)**		***p*-value**	**Statistics**
	**Mean (SD)**	**(%) (range)**	**Mean (SD)**	**(%) (range)**		
Age	12(5.6)	[6–28]	12(4.8)	[6–27]	*p* = 0.78	z = 0.28
Gender (male)		7 (37%)		16 (67%)	0.052	χ^2^ = 3.8
Estimated IQ	68 (31)	[20–112]	110 (12)	(97–134)	*P* < 0.001***	z = 4.4

**Table 2:** Clinical features of the subjects with TSC (*n* = 19).

**No**	**Age/year**	**Sex**	**ASD**	**IQ**	**Epilepsy type**	**Onset/months**	**Epilepsy severity/frequency**	**Tuber location**	**mTOR inhibitors**	**SRS-T score**	**Vlad MB/SO score**
1	6	F	Yes	33	DEE/FE	9	1	Multi/RL	No	102	22/38
2	7	F	Yes	23	DEE/FE	3	2/w	Multi/RL	No	80	19/20
3	7	F	No	112	FE	33	1	Multi/RL	No	53	18/72
4	7	M	Yes	25	DEE	5	1	Multi/RL	No	99	21/28
5	8	F	No	82	FE	8	1	Multi/RL	No	45	18/80
6	9	F	No	73	DEE	5	1	Multi/RL	No	47	17/80
7	9	M	No	43	DEE/FE	9	2/m	Multi/RL	Yes-1	76	17/45
8	10	M	No	96	NA	108	1	Multi/L	No	53	15/69
9	10	M	No	80	NA	NA	0	Multi/RL	No	53	16/73
10	11	M	No	100	FE	96	1	Multi/RL	No	54	15/85
11	12	F	No	105	NA	NA	0	Multi/L	No	68	19/71
12	13	F	Yes	103	NA	NA	0	Multi/RL	No	83	19/88
13	13	F	Yes	61	FE	65	2/m	Multi/RL	Yes-1	80	20/69
14	14	F	No	53	NA	NA	0	Multi/RL	No	68	18/56
15	14	M	Yes	45	FE	18	1	Multi/RL	No	86	21/37
16	15	M	No	88	FE	60	1	Multi/RL	No	61	16/77
17	15	F	No	105	NA	NA	0	Multi/RL	Yes-2	63	20/72
18	23	F	Yes	49	FE	28	2/m	Multi/RL	Yes-1	NA	20/38
19	28	F	Yes	20	DEE/FE	6	2/w	Multi/RL	Yes-1	NA	NA/20

*M, male; F, female; DEE, developmental and epileptic encephalopathies; FE, focal epilepsy; NA, not available; onset/months, epilepsy onset in months; epilepsy severity: 0, never; 1, well controlled (seizure-free for more than a year); 2, refractory epilepsy; w, weekly; m, monthly; multi, multiple cortical tubers; RL, in both hemisphere (right and left); L, in left hemisphere; mTOR inhibitors, yes-1_for refractory epilepsy and yes-2 for subependymal giant cell astrocytoma (SEGA); Vlad MB/SO score, vineland maladaptive behavior score/socialization score*.

### Imaging Results

#### TBSS Results

**Figure 2:**
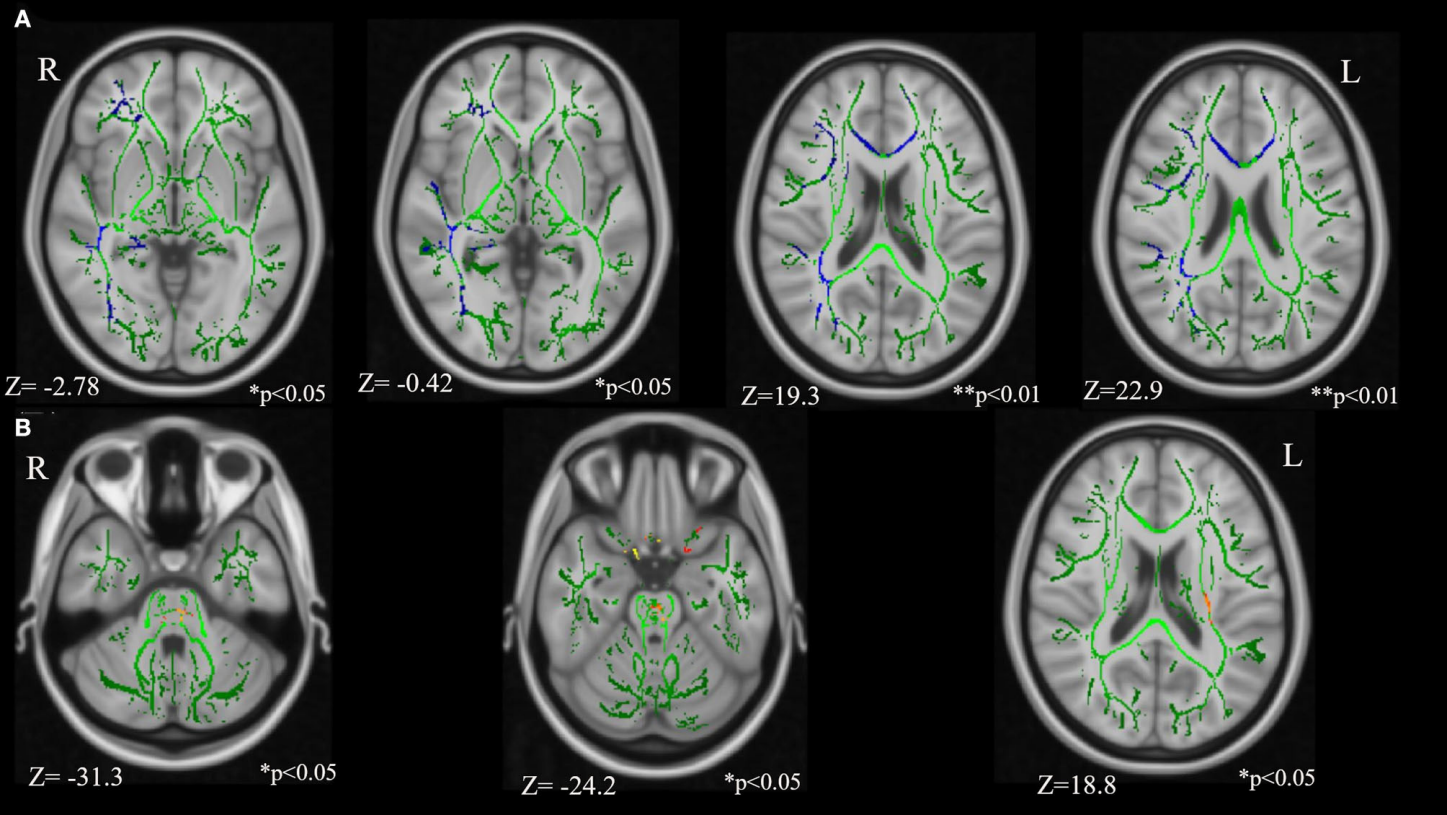
Results of the tract-based spatial statistics revealed significant differences between the TSC and control groups in fractional anisotropy (FA). **(A)** Axial slices of mean FA skeleton (green) represent areas of no significant difference between groups. Regions in blue indicate areas where the value of FA is significantly lower in the TSC group (TSC < Control); right IFOF**, right SLF**, genu of CC*, right UF*, and right ILF*. **(B)** Regions in yellow/orange/red indicate areas where the value of FA is significantly higher in the TSC group (TSC > Control); pontine crossing tract*, bilateral CST*. FWE corrected, *p* < 0.05, with a cluster size of voxels > 10. Results are adjusted for age and sex. **p < 0.001, *p < 0.05. IFOF, inferior frontal occipital fasciculus; SLF, superior longitudinal fasciculus; ILF, inferior longitudinal fasciculus; UF, uncinate fasciculus; CST, corticospinal tract; FWE, family wise error.

Comparison between the TSC and control groups revealed statistically significant group differences (Figure 2; Table 3). FA in the right IFOF, right ILF, right SLF, genu of CC, and right UF were significantly lower (FWE corrected *p* < 0.05, results were controlled for age and sex) in the TSC group than in the control group (Figure 2A). In contrast, FA in the pontine crossing tract and bilateral corticospinal tract (CST) were significantly higher (FWE corrected *p* < 0.05, results were controlled for age and sex) in the TSC group than in the control group (Figure 2B).

**Table 3:** The regions showing significant FA differences between TSC and control group.

**Anatomical region**	**Side**	**Cluster**	**Cluster**	**MNI coordinates, mm**		
		**Size**	***p*-value**	**COGX**	**COGY**	**COGZ**
**Control group** **>** **TSC group**						
Inferior front-occipital fasciculus	R	7,059	0.004	30.9	−40.9	19.3
Superior longitudinal fasciculus	R	5,602	0.004	32.2	−42.5	22.9
Genu of corpus callosum	R	2,071	0.013	−1.7	22.8	18.9
Uncinate fasciculus	R	120	0.038	30.1	44	−2.78
Inferior longitudinal fasciculus	R	31	0.033	35.2	−68.6	−0.42
**TSC group** **>** **control group**						
Pontine crossing tract		469	0.012	−1.67	−26.6	−24.2
Corticospinal tract	L	270	0.02	−26.9	−23.3	18.8
Corticospinal tract	R	29	0.038	5.6	−30.2	−31.3

*All regions showing significant difference at p < 0.05 using a family-wise error (FWE) rate correction with a cluster size of voxel > 10. Results were controlled for age and sex*.

#### TAND-Related White Matter in Subjects With TSC (n = 19)

**Figure 3:**
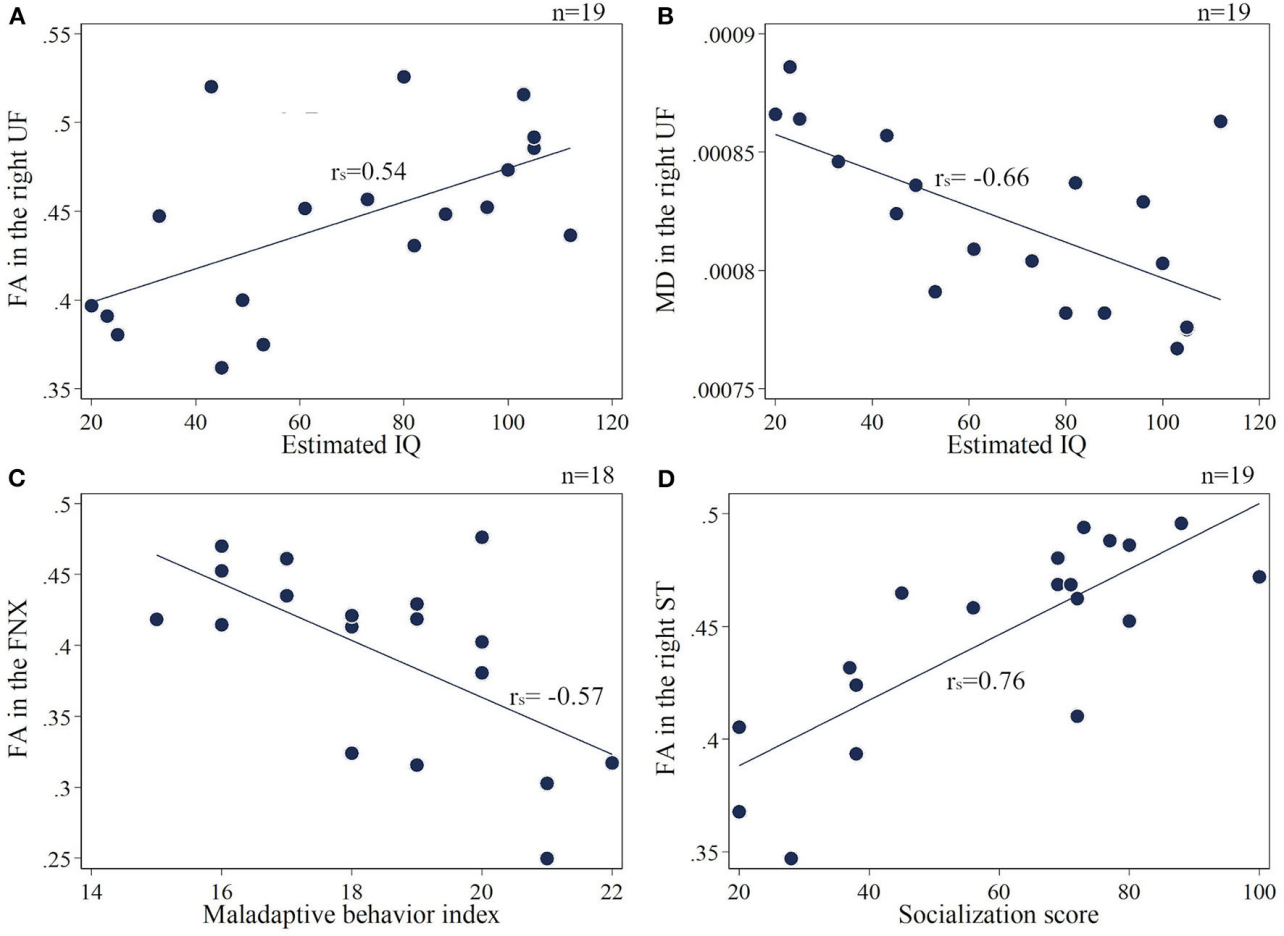
**(A,B)** Correlation of the estimated IQ and FA/MD in the right UF. **(C)** Maladaptive behavior index negatively correlates with FA in the FNX. **(D)** Socialization score positively correlates with FA in the right ST. UF, uncinate fasciculus; FNX, body of the fornix; ST, stria terminalis.

The extracted mean FA/MD values are shown in the Supplementary Materials 2–3. The association between four assessment scales of TAND (estimated IQ, socialization score, maladaptive behavior index, and SRS-T score) and FA/MD in seven target regions: (1) FNX, (2) SS, (3) ST, (4) CGC, (5) SLF, (6) UF, and (7) CC, were extracted. The estimated IQ scores showed a significantly positive correlation with FA (rs = 0.54, *p* = 0.017) and a negative correlation with MD (rs = −0.66, *p* = 0.002) in the right UF (Figures 3A,B). In addition, FA in the bilateral SS (right: rs = 0.72, *p* < 0.001; left: rs = 0.57, *p* = 0.01), splenium of the CC (rs = 0.64, *p* = 0.004), bilateral ST (right: rs = 0.59, *p* = 0.008; left: rs = 0.65, *p* = 0.003), and right SLF (rs = 0.52, *p* = 0.02) showed a positive correlation with estimated IQ. MD in the right SS (rs = −0.56, *p* = 0.011), bilateral SLF (right: rs = −0.78, *p* < 0.001; left: rs = −0.53, *p* = 0.021), and bilateral CGC (right: rs = −0.68, *p* = 0.001; left: rs = −0.67, *p* = 0.002) showed a negative correlation with estimated IQ. The Vineland Maladaptive Behavior Index was negatively correlated with FA in the FNX (rs = −0.57, *p* = 0.013; Figure 3C). Meanwhile, The Vineland socialization score was positively correlated with FA in the right UF (rs = 0.62, *p* = 0.005), splenium of CC (rs = 0.81, *p* < 0.001), bilateral ST (right: rs = 0.76, *p* < 0.001; left: rs = 0.73, *p* < 0.001; Figure 3D), bilateral SS (right: rs = 0.86, *p* < 0.001; left: rs = 0.75, *p* < 0.001), and FNX (rs = 0.58, *p* = 0.009), whereas it was negatively correlated with MD in the splenium of CC (rs = −0.63, *p* = 0.004), bilateral ST (right: rs = −0.48, *p* = 0.04; left: rs = −0.5, *p* = 0.03), bilateral SLF (right: rs = −0.71, p < 0.001; left: rs = −0.62, *p* = 0.004), bilateral CGC (right: rs = −0.63, *p* = 0.004; left: rs = −0.64, *p* = 0.003), right UF (rs = −0.67, *p* = 0.002), right SS (rs = −0.59, *p* = 0.008), and FNX (rs = −0.46, *p* = 0.046). The SRS-T score has a negative correlation with FA (rs = −0.54, *p* = 0.025) and positive correlation with MD (rs = 0.5, *p* = 0.04) in the splenium of the CC.

**Figure 4:**
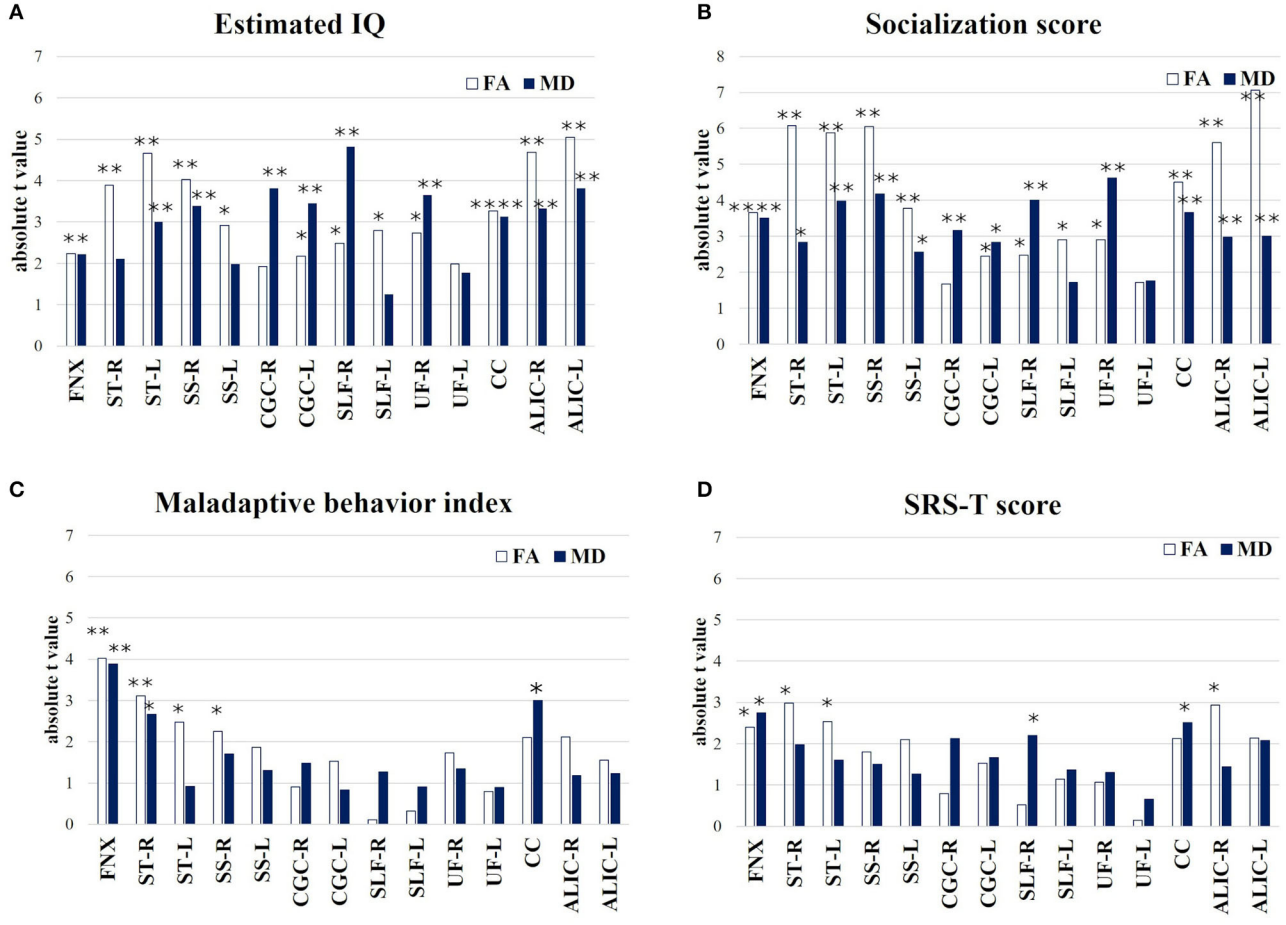
Multiple linear regression (absolute *t*-value) indicates the strength of association between behavior assessment **(A)** Estimated IQ; **(B)** Socialization score; **(C)** Maladaptive behavior index; **(D)** SRS-T score and FA/MD in target 7 regions and ALIC. *p < 0.05, **p < 0.01. FNX, body of the fornix; ST, stria terminalis; SS, sagittal stratum; CGC, cingulum in the cingulate gyrus; SLF, superior longitudinal fasciculus; UF, uncinate fasciculus; CC, splenium of the corpus callosum; ALIC, anterior limb of internal capsule.

The results of the multiple linear regression, where age and sex were included as covariates and their effects were adjusted for in a linear regression model, are shown in the Supplementary Materials 4–7. The absolute *t*-value indicates the strength of the association between the four assessment scales of TAND and FA/MD in seven target regions and ALIC (Figure 4). The results of the multiple linear regression analysis between refractory epilepsy and FA/MD in the seven target regions are summarized in Supplementary Material 8. Estimated IQ widely affected both FA and MD in all seven target regions, particularly the long association fibers, such as the following: FA/MD in the right SLF (β = 4.41 × 10^−4^, *p* = 0.03, *t* = 2.48/β = −1.09 × 10^−6^, *p* < 0.001, *t* = −4.82), right SS (β = 7.5 × 10^−4^, *p* = 0.001, *t* = 4.03/β = −1.81 × 10^−6^, *p* = 0.004, *t* = −3.39, Figure 4A).

The socialization score also affected both FA and MD in the seven target regions; such as the right UF (β = 1.27 × 10^−3^, *p* = 0.011, *t* = 2.9/β = −1.2 × 10^−6^, *p* < 0.001, *t* = −4.62, Figure 4B) and splenium of CC (β = 1.22 × 10^−3^, *p* < 0.001, *t* = 4.51/β = −2.3 × 10^−6^, *p* = 0.002, *t* = −3.66).

The Maladaptive Behavior Index significantly affected both FA/MD in the FNX (β = −2.47 × 10^−2^, *p* = 0.001, *t* = −4.02/β = 1.2 × 10^−4^, *p* = 0.002, *t* = 3.89) and right ST (β = −1.44 × 10^−2^, *p* = 0.008, *t* = −3.11/β = 4.2 × 10^−5^, *p* = 0.019, *t* = 2.66, Figure 4C).

The SRS-T score also affected both FA/MD in the FNX (β = −1.9 × 10^−3^, *p* = 0.032, *t* = −2.4/β = 1.03 × 10^−5^ *p* = 0.017, *t* = 2.75, Figure 4D).

**Figure 5:**
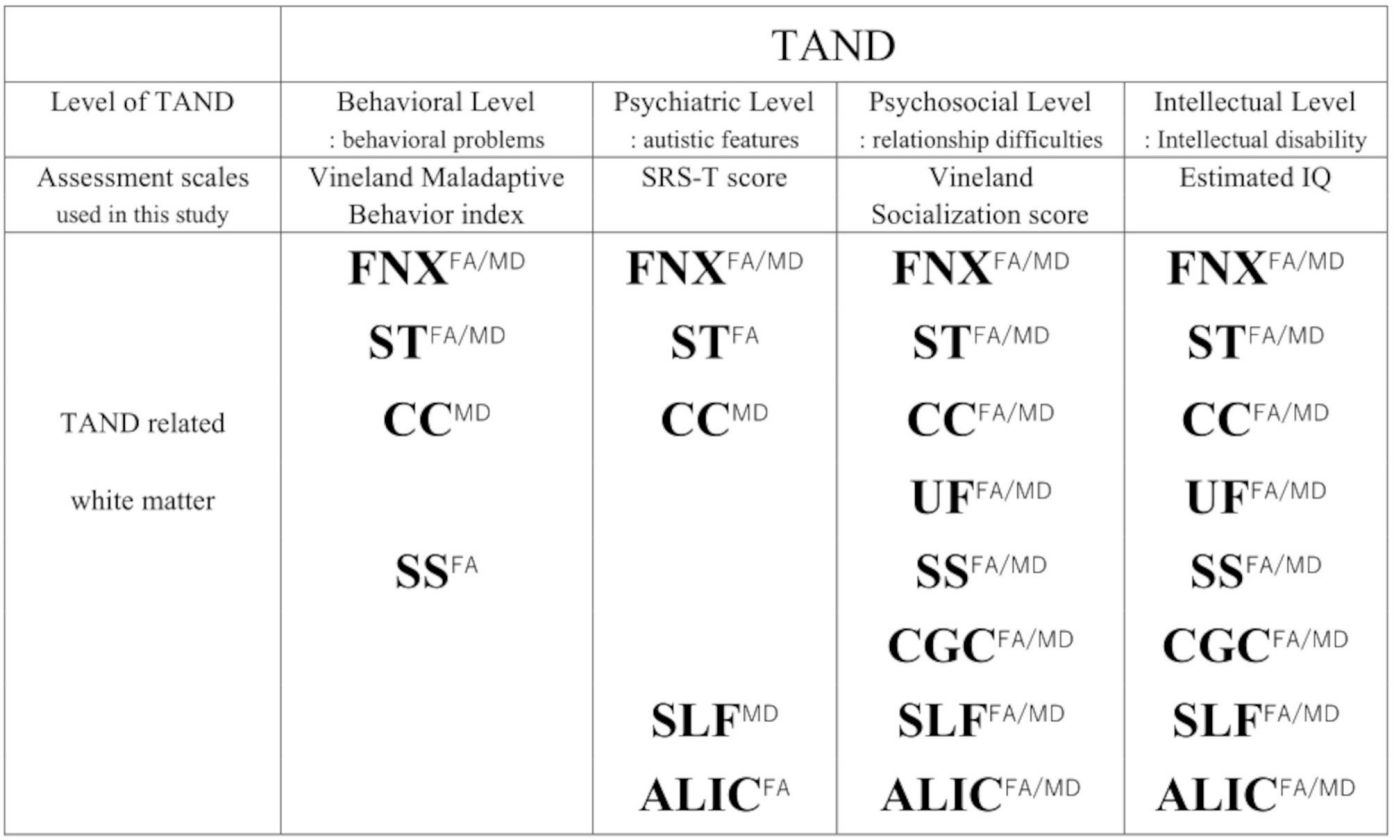
Summary of TAND and related white matter depending on each TAND level. To assess TAND manifestations, Estimated IQ, Socialization score, Maladaptive Behavior Index, SRS-T score are used. Superscripts (FA/MD, FA, MD) means both FA and MD, FA, MD.FNX, body of the fornix; ST, stria terminalis; SS, sagittal stratum; CGC, cingulum in the cingulate gyrus; SLF, superior longitudinal fasciculus; UF, uncinate fasciculus; CC, splenium of the corpus callosum; ALIC, anterior limb of internal capsule.

The four levels of TAND used in this study and the relevant white matter tracts depending on each TAND level (8), except for the academic and neuropsychological levels, are summarized in Figure 5. In addition, estimated IQ and socialization score strongly affected both FA/MD in the bilateral ALIC (estimated IQ; FA/right; β = 7.5 × 10^−4^, *p* < 0.001, *t* = 4.68, left; β = 7.7 × 10^−4^, *p* < 0.001, *t* = 5.05, MD/right; −7.2 × 10^−7^, *p* = 0.005, *t* = 3.3, *left*; β = 8.0 × 10^−7^, *p* = 0.002, *t* = −3.8, socialization score; FA/right; β = 1.1 × 10^−3^, *p* < 0.001, *t* = 5.6, left; β = 1.1 × 10^−3^, *p* < 0.001, *t* = 7.1, *MD*/*right*; 9.0 × 10^−7^, *p* = 0.009, *t* = 3.0, *left*; β = 9.3 × 10^−7^, *p* = 0.009, *t* = 3.0). The SRS-T score also affected FA in the right ALIC (β = −8.3 × 10^−4^, *p* = 0.012, *t* = −2.9).

Refractory epilepsy affected both FA/MD in the left ST (β = −5.7 × 10^−2^, *p* = 0.03, *t* = −2.4/β = 1.68 × 10^−4^, *p* = 0.002, *t* = 3.82), right SLF (β = −3.3 × 10^−2^, *p* = 0.043, *t* = −2.21/β = 8.0 × 10^−5^, *p* = 0.002, *t* = 3.72), right SS (β = −4.5 × 10^−2^, *p* = 0.03, *t* = −2.4/β = 1.38 × 10^−4^, *p* = 0.009, *t* = 2.98), FA in the left SS (β = −4.8 × 10^−2^, *p* = 0.016, *t* = −2.7) and left SLF (β = −3.0 × 10^−2^, *p* = 0.028, *t* = −2.43), MD in the bilateral CGC (right; β = 4.0 × 10^−5^, *p* = 0.022, *t* = 2.55, left; β = 4.5 × 10^−5^, *p* = 0.04, *t* = 2.25), and right UF (β = 6.9 × 10^−5^, *p* = 0.001, *t* = 3.93).

The Mann-Whitney U test did not show significant differences between the TSC subjects using mTOR inhibitors (*n* = 5) and those not using mTOR inhibitors (*n* = 14) in terms of FA/ MD in all the seven target regions.

## Discussion

Our results suggest that TANDs are not the result of an abnormality of specific brain region, but rather caused by connectivity dysfunction as a network disorder. Association analysis showed that Intellectual level (ID) was widely associated with all seven target regions and ALIC, and was strongly associated with long association fibers such as the SLF, SS (IFOF + ILF) and UF in the right hemisphere. These tracts overlayed with the results of the TBSS which showed decreased FA in subjects with TSC. The IFOF is supposed to play an important role in the frontal-subcortical circuits (42) and the UF connects the OFC with the anterior temporal lobes, and the abnormality of these white matter tracts is associated with psychiatric disorders (24, 42). In this study, the behavioral level (behavioral problems) and psychiatric level (autistic features) were associated with the limbic system, including the FNX and ST, and CC. Furthermore, the right ALIC was associated with the autistic features. These findings may reflect disrupted functional connectivity of white matter (3). The disruption of white matter integrity may induce underconnectivity between cortical and subcortical regions, which is relevant to psychiatric symptoms (43). This is the first report showing that abnormal white matter connectivity including the limbic system is associated with TAND.

### Pathophysiological Mechanism of Neurological Manifestations in TSC

In the TSC brain, there are two main pathophysiological mechanisms for neurological manifestations: (1) focal abnormalities associated with cortical tubers, and (2) abnormal structural and functional connectivity.

Cortical tubers are lesions with cortical malformations, which result from the abnormal maturation and differentiation of neurons (44). These morphological changes originate from the overactivation of the mTOR pathway in the early stage of development. Dysmorphic neurons in the cortical tubers cause neurological dysfunction. Recently, fetal cerebral lesion scores have been reported to correlate with neurodevelopment and ASD in TSC (45). Therefore, TAND manifestations and microstructural disruption could result from cortical malformations. Cortical tubers can also induce epilepsy (46). Complete resection of the epileptogenic zone associated with cortical tubers has been successfully reported in TSC (47). IS, early-onset epilepsy, and refractory epilepsy were reported to be risk factors for TAND (12, 48, 49). In this study, IS and early-onset epilepsy were risk factors for ID but were not risk factors for ASD features. Studies suggested that severe or uncontrolled epilepsy cause poor cognitive outcomes (50, 51). Here, refractory epilepsy was associated with both FA and MD in the left ST, right SLF, right SS, FA in the left SS, SLF, MD in the bilateral CGC, and right UF. These findings coincide with a DTI study of children with TSC with persistent seizures (49). Our results suggest that refractory epilepsy influences the alternation of the white matter microstructure integrity in the frontal and temporal areas, which are strongly associated with intellectual ability and sociability. In contrast, the effect of epilepsy on ASD is unclear and requires further exploration (50–52). It is important to consider that the adverse effect of treatment of epilepsy affects the neuro-psychiatric manifestations in subjects with TSC (53). In this study, we did not compare the location or volume of cortical tubers with DTI parameters, because most of the patients showed multiple cortical tubers and had poorly defined borders. TSCs have been reported to exhibit abnormalities in structural and functional connectivity. Many animal studies have demonstrated the crucial role of the mTOR pathway, especially mTORC1, in oligodendrocyte differentiation (54, 55) and myelin gene expression (56, 57). A mouse model with oligodendrocyte-specific overactivation of mTORC1 showed decreased p4E-BP1, pAKT, and hypomyelination (56). These data suggest that mTORC1 activity disrupts myelination. A recent neuropathologic study reported a significant association between IQ at surgery and reduced numbers of oligodendroglia in the white matter of cortical tubers in subjects with TSC (58). Increased MD and decreased FA suggest abnormal myelination or disrupted microstructural organization of the white matter in subjects with TSC (3). In addition, two event-related potential (ERP) studies have reported atypical face processing in an individual with TSC (59, 60). The longer latency of ERPs indicated slower face processing, which suggested a disruption of myelination or weakness in neural connectivity.

### Abnormal White Matter Development in ASD and TSC

The cumulative abnormalities in white matter microstructural integrity in the corpus callosum (CC) were associated with increasing neurological comorbidities, such as ID, epilepsy, and ASD in subjects with TSC (15), and the comorbid diagnosis of ASD was associated with the largest change in FA (15). Several studies have reported that there was a significant difference in the microstructural integrity of the CC between children with idiopathic ASD and healthy controls, and the DTI parameters of CC were correlated with the socio-communicative ability (61, 62). In addition, significant differences in FA in the arcuate fasciculus between TSC subjects with and without ASD have been reported (63). A longitudinal study of infants with TSC (0–2 years old) revealed that subjects with TSC who develop ASD exhibit reduced FA in several major white matter tracts, including the arcuate fasciculus, CC, and the SS, relative to TSC subjects without ASD (64). These findings suggest abnormal myelination or disrupted microstructural organization of white matter in the developing brain. This study showed significantly reduced FA in the right association fibers such as the IFOF, ILF, SLF, UF, and CC in the TSC group compared with the healthy control group. The IFOF connects the occipital lobe and OFC. The short fibers of the ILF connect the occipital lobe and temporal lobe, and the long fibers of the ILF connect the visual areas of the occipital lobe to the amygdala and hippocampus (65). The IFOF and ILF have been repeatedly reported to be critically important for face processing (66). The SLF connects the parietal, occipital, and temporal lobes (66), and it is related to the major cognitive abilities, such as language, attention, and memory; in particular, the right SLF is associated with visual perception (67, 68). Furthermore, the OFC is important for the recognition of expressed emotions (69). The UF connects the temporo-amygdala-orbitofrontal network (22); in particular, the right UF is strongly associated with the emotional empathy network (23). Therefore, we hypothesize that reduced FA in the right association fibers in subjects with TSC indicates socio-emotional difficulties such as a lack of emotional empathy, poor face processing, or unrecognition of expressed emotion. These behavioral features may be considered as the parts of TAND. On the other hand, FA in the pontine crossing tract and bilateral CST were significantly higher in the TSC group than in the control group. A limitation of DTI is the difficulty in representing crossing white matter fascicles (70). Generally, crossing fiber problems occur when there are more than two fiber bundles that are differently oriented in the same voxel (71), such as SLF and CST, which are crossing in centrum semiovale (72, 73). FA is sensitive to microstructural change (74), and FA decrease reflects the reduced white matter integrity. However, where nerves cross, an atypical increase in FA may occur owing to a higher apparent diffusion coefficient of the other nerve when one nerve is damaged (75). For example, DTI studies of subjects with both multiple sclerosis (MS) and Alzheimer's diseases reported a higher FA than healthy controls in the crossing fiber regions when one of the fiber bundles was degenerated (75, 76).

Here, TBSS showed a higher FA in the bilateral CST and pontine crossing tract, which are the crossing fiber regions. We speculate that one damaged fiber bundle in the crossing fiber regions may induce an atypical increase in FA in subjects with TSC.

### Limbic System and TAND Manifestation

Bolton et al. (77) reported that developmental abnormalities in the temporal lobe, resulting from temporal tubers could be a risk factor for comorbidity with ASD, because of impairments in visual recognition, such as facial expression. “The amygdala theory of autism” proposed that abnormalities in the amygdala could be a cause of autism, which suggested that three regions, 1 the amygdala, 2 the OFC, and 3 the superior temporal sulcus and gyrus, play an important role in social intelligence (78, 79). The TAND psychiatric level includes ASD, ADHD, anxiety disorders, and depressive disorders (9). In this study, the SRS-T score, which represents autistic features, was associated with FA and MD in the FNX, FA in the bilateral ST and right ALIC, and MD in the splenium of the CC. A previous mega analysis DTI study of psychiatric disorders revealed that there was a common white matter alternation in subjects with schizophrenia and bipolar disorder in the limbic system, including the fornix and cingulum (80). The hippocampus and the mammillary body are connected by the body of the fornix, and the mammillary body and anterior nuclei are connected by the mammillothalamic tract, which forms the hippocampal-diencephalic-cingulate network associated with memory, emotion, and psychiatry (21, 22). The ST is a small limbic pathway that forms the main efferent pathways from the amygdala, and run parallel to the fornix (21). The ST combines the amygdala with the bed nucleus of stria terminalis (BNST) (81). The BNST is the hub for integration of limbic information and contains many sub-nuclei. The BNST is important for detection and expression of fear and, regulates emotional state or arousal (82). All the prefrontal cortex fibers (both ascending and descending) are projecting to the ALIC, which is one of the most established targets of deep brain stimulation (DBS) or other neuro-surgical treatment for neuropsychiatric disorders (26, 83). The dysfunction of the cortico-striato-thalamo-cortical (CSTC) loop is common model for neuropsychiatric disorders (84). Recently, the effects of DBS to the ALIC, the nucleus accumbens (NAcc), basolateral amygdala, and hypothalamus for self-injury behavior in subjects with ASD were reported (85, 86), and these areas are in anatomical proximity with the targets of DBS for obsessive compulsive disorder(OCD) (87). In this study, TAND behavioral level difficulty was associated with FNX and ST, and the ALIC was associated with autistic features. These results suggest the existence of aberrant neural network, which may be relevant to behavioral problems, such as anxiety, severe aggression, or self-injury, in subjects with TSC, as well as other neuropsychiatric disorders.

### Future Directions of DTI Studies in TSC

Recently, mTOR-inhibitors, that block mTORC-1 have been approved for the manifestations of TSC, such as renal angiomyolipoma, SEGAs, and epilepsy (4, 88, 89). Although mTOR-inhibitors show definite efficacy in epilepsy, their effectiveness in TAND remains controversial (88, 90, 91). In a human study, primary mixed glial cell cultures derived from patients with TSC or focal cortical dysplasia, which is a histologically close condition, reduced oligodendroglial turnover in association with a lower myelin content. In addition, mTOR inhibitors repair decreased myelination (92). Two studies have reported that mTOR inhibitors change the white matter microstructure (88, 89). The efficacy of everolimus for intractable epilepsy and the improvement of autistic features in patients with TSC have been reported in a previous Japanese study (90). In this study, five subjects with TSC (26%) were treated with an mTOR-inhibitor (four had refractory epilepsy, one was the treatment of SEGA). Although we considered the effect of mTOR inhibitors on white matter integrity, we included these patients because this study focused on the association between TAND manifestations and white matter microstructure regardless of treatment. As a result, the FA value of major white matter tracts was not high in the subjects using mTOR inhibitor (Supplementary Material 8). To evaluate the effects of mTOR-inhibitors on white matter microstructure, we should observe the change in DTI parameters before and after treatment with mTOR-inhibitors. In the future, the effect of mTOR-inhibitors on white matter microstructure associated with TAND manifestations should be examined in longitudinal studies with a large cohort. Particularly, investigating whether the change in behavior score coincides with changes in the limbic white matter and neural networks may reveal the effects of mTOR-inhibitors on TAND.

### Limitations

The present study has several limitations. First, our sample size was small, and the study design was not longitudinal. When we consider the TAND as a neural network disorder, TBSS is not adequate to identify specific neural network to basal ganglia and/ or brainstem, which recently got attention for the association with neuropsychiatric disorders. The future studies with a large cohort and advanced neuro-imaging techniques are required. Longitudinal follow-up is needed to confirm and extend our results. Second, the effects of genotype were not considered. Since we did not obtain genetic information, we could not consider any genotype-phenotype correlations. As a previous study reported genotype-TAND correlations in TSC2 (9), the correlation of genotype should be performed in the future. Third, we did not consider the effect of tubers on DTI analysis. In the future, positron emission tomography should be studied to reveal the effect of tubers on TAND (81).

## Conclusion

This study indicates that abnormal white matter connectivity including the limbic system is associated with TAND. Our results suggest that TANDs are not the result of an abnormality of specific brain region, but rather caused by connectivity dysfunction as a network disorder.

This study highlights the importance of analyzing the relationship between brain and behavior to reveal TAND related white matter and networks.

## Data Availability Statement

The original contributions presented in the study are included in the article/Supplementary Material, further inquiries can be directed to the corresponding author/s.

## Ethics Statement

The studies involving human participants were reviewed and approved by Institutional Review Board of Osaka University Hospital (No. 18491). Written informed consent to participate in this study was provided by the participants' legal guardian/next of kin.

## Author Contributions

AS contributed to the design of study, the data collection and data analysis, and drafted the manuscript. KT, MW-K, and YK contributed to the data collection. KT, YI, and MT contributed to the analysis and interpretation of the data and revised the manuscript. KM and KN contributed to the DTI analysis. KK-S supervised and directed this study. All authors have read and approved this manuscript submission.

## Funding

This study was supported by JSPS KAKENHI (Grant Numbers: JP21K12153 and JP18K07843) and the AMED (Grant Number: dm0307103h0003).

## Conflict of Interest

The authors declare that the research was conducted in the absence of any commercial or financial relationships that could be construed as a potential conflict of interest.

## Publisher's Note

All claims expressed in this article are solely those of the authors and do not necessarily represent those of their affiliated organizations, or those of the publisher, the editors and the reviewers. Any product that may be evaluated in this article, or claim that may be made by its manufacturer, is not guaranteed or endorsed by the publisher.
