# Supplementary material for: Abnormal White Matter Microstructure in the Limbic System Is Associated With Tuberous Sclerosis Complex-Associated Neuropsychiatric Disorders
Source: Front Neurol. 2022 Mar 14;13:782479. doi: 10.3389/fneur.2022.782479 (PMC8963953; doi:10.3389/fneur.2022.782479)
Supplement: Supplementary file 1 [file Data_Sheet_1.docx]

**Supplementary material 1** | The type and rate of TAND behavioral level difficulties in subjects with TSC, assessed by TAND check list (n=19)

| anxiety | 8 (42%) |
| --- | --- |
| tantrum | 6 (32%) |
| severe aggression | 4 (21%) |
| impulsivity | 4 (21%) |
| overactivity | 3 (16%) |
| self- injury | 3 (16%) |
| sleep difficulty | 3 (16%) |
| depression | 2 (11%) |

**Supplementary material 2** | The extracted mean FA value of target 7 regions and ALIC in 19 subjects with TSC

| No | CC | FNX | SS-R | SS-L | CGC-R | CGC-L | ST-R | ST-L | SLF-R | SLF-L | UF-R | UF-L | ALIC-R | ALIC-L |
| --- | --- | --- | --- | --- | --- | --- | --- | --- | --- | --- | --- | --- | --- | --- |
| 1 | 0.653635 | 0.317143 | 0.454531 | 0.394713 | 0.340887 | 0.348826 | 0.393458 | 0.438086 | 0.384621 | 0.362403 | 0.447328 | 0.437048 | 0.465696 | 0.446057 |
| 2 | 0.612091 | 0.315641 | 0.413913 | 0.381086 | 0.339035 | 0.370743 | 0.367746 | 0.365661 | 0.399731 | 0.360502 | 0.390936 | 0.414876 | 0.450201 | 0.408079 |
| 3 | 0.682491 | 0.324012 | 0.457471 | 0.433128 | 0.373358 | 0.413632 | 0.410153 | 0.465157 | 0.39435 | 0.406039 | 0.436465 | 0.400475 | 0.522626 | 0.502273 |
| 4 | 0.681719 | 0.249729 | 0.453992 | 0.431735 | 0.354837 | 0.375238 | 0.346964 | 0.377894 | 0.419343 | 0.420833 | 0.380412 | 0.380511 | 0.479403 | 0.467112 |
| 5 | 0.719268 | 0.421051 | 0.500258 | 0.456558 | 0.392942 | 0.423652 | 0.486129 | 0.473784 | 0.423232 | 0.426781 | 0.43066 | 0.371975 | 0.51046 | 0.509062 |
| 6 | 0.731158 | 0.435019 | 0.494651 | 0.472013 | 0.394593 | 0.4327 | 0.452403 | 0.499447 | 0.420101 | 0.412974 | 0.456709 | 0.439073 | 0.51511 | 0.503852 |
| 7 | 0.682121 | 0.461178 | 0.473517 | 0.427127 | 0.382605 | 0.412566 | 0.464816 | 0.465428 | 0.385874 | 0.395716 | 0.520229 | 0.481206 | 0.520589 | 0.494038 |
| 8 | 0.674378 | 0.418377 | 0.473946 | 0.407583 | 0.36104 | 0.392396 | 0.468628 | 0.460117 | 0.399227 | 0.392361 | 0.452275 | 0.430563 | 0.497904 | 0.481118 |
| 9 | 0.734816 | 0.452546 | 0.511354 | 0.479826 | 0.384194 | 0.430153 | 0.493997 | 0.520117 | 0.453502 | 0.432614 | 0.525786 | 0.478349 | 0.54723 | 0.527819 |
| 10 | 0.692932 | 0.414607 | 0.494927 | 0.433133 | 0.334286 | 0.40151 | 0.472037 | 0.470845 | 0.415842 | 0.418852 | 0.473314 | 0.43147 | 0.525255 | 0.5205 |
| 11 | 0.727173 | 0.429245 | 0.505904 | 0.460571 | 0.497471 | 0.513284 | 0.468583 | 0.52039 | 0.446971 | 0.428772 | 0.491727 | 0.491496 | 0.514641 | 0.500963 |
| 12 | 0.722914 | 0.418644 | 0.536482 | 0.46897 | 0.380054 | 0.407393 | 0.495797 | 0.507196 | 0.44117 | 0.435345 | 0.515814 | 0.476573 | 0.528336 | 0.535238 |
| 13 | 0.702422 | 0.476286 | 0.480168 | 0.430105 | 0.430599 | 0.501564 | 0.480373 | 0.49141 | 0.428927 | 0.411352 | 0.451606 | 0.442167 | 0.510815 | 0.515208 |
| 14 | 0.66646 | 0.413133 | 0.4758 | 0.461816 | 0.408729 | 0.430293 | 0.458309 | 0.463048 | 0.366513 | 0.372486 | 0.374866 | 0.334051 | 0.492594 | 0.470598 |
| 15 | 0.607954 | 0.302686 | 0.420322 | 0.410769 | 0.358785 | 0.3739 | 0.431663 | 0.436578 | 0.426715 | 0.425992 | 0.361835 | 0.375194 | 0.484647 | 0.484796 |
| 16 | 0.712959 | 0.470011 | 0.511933 | 0.500223 | 0.425894 | 0.442866 | 0.488117 | 0.511765 | 0.443097 | 0.43311 | 0.448407 | 0.443319 | 0.530634 | 0.51615 |
| 17 | 0.710528 | 0.380636 | 0.500727 | 0.467612 | 0.408685 | 0.437138 | 0.462402 | 0.495642 | 0.430898 | 0.418678 | 0.485561 | 0.463051 | 0.527446 | 0.521811 |
| 18 | 0.647837 | 0.402469 | 0.450766 | 0.412259 | 0.379556 | 0.38655 | 0.42398 | 0.474085 | 0.393762 | 0.402449 | 0.399948 | 0.400026 | 0.418158 | 0.494794 |
| 19 | 0.66272 | 0.346132 | 0.449128 | 0.414416 | 0.376968 | 0.430507 | 0.405303 | 0.456587 | 0.363962 | 0.419409 | 0.396787 | 0.352796 | 0.444395 | 0.477907 |

CC, splenium of the corpus callosum; FNX, body of the fornix; SS, sagittal stratum; CGC, cingulum in the cingulate gyrus;

ST, stria terminalis; SLF, superior longitudinal fasciculus; UF, uncinate fasciculus; ALIC, anterior limb of internal capsule; R, right; L, left.

**Supplementary material 3** | The extracted mean MD value of target 7 regions and ALIC in 19 subjects with TSC

| No | CC | FNX | SS-R | SS-L | CGC-R | CGC-L | ST-R | ST-L | SLF-R | SLF-L | UF-R | UF-L | ALIC-R | ALIC-L |
| --- | --- | --- | --- | --- | --- | --- | --- | --- | --- | --- | --- | --- | --- | --- |
| 1 | 0.000954 | 0.00176 | 0.001015 | 0.001051 | 0.000839 | 0.000839 | 0.001048 | 0.000981 | 0.000854 | 0.000903 | 0.000846 | 0.000848 | 0.000743 | 0.000783 |
| 2 | 0.001036 | 0.002009 | 0.001183 | 0.001471 | 0.000838 | 0.000853 | 0.001097 | 0.001258 | 0.000827 | 0.000872 | 0.000886 | 0.000882 | 0.000797 | 0.00084 |
| 3 | 0.000827 | 0.001766 | 0.000996 | 0.000979 | 0.00074 | 0.000754 | 0.001147 | 0.000994 | 0.000762 | 0.000756 | 0.000863 | 0.000815 | 0.000664 | 0.000664 |
| 4 | 0.000972 | 0.002682 | 0.000986 | 0.001128 | 0.00079 | 0.00082 | 0.001489 | 0.001046 | 0.000822 | 0.000801 | 0.000864 | 0.000831 | 0.000745 | 0.000763 |
| 5 | 0.000842 | 0.001377 | 0.000898 | 0.00097 | 0.000781 | 0.000773 | 0.000924 | 0.000829 | 0.000776 | 0.000768 | 0.000837 | 0.000876 | 0.000725 | 0.000731 |
| 6 | 0.000854 | 0.001461 | 0.000974 | 0.00095 | 0.000793 | 0.000789 | 0.000952 | 0.000891 | 0.000789 | 0.000796 | 0.000804 | 0.00078 | 0.000757 | 0.000773 |
| 7 | 0.000839 | 0.001373 | 0.000962 | 0.000916 | 0.000833 | 0.00082 | 0.000881 | 0.001008 | 0.000853 | 0.000845 | 0.000857 | 0.000807 | 0.000743 | 0.000756 |
| 8 | 0.000914 | 0.00165 | 0.000934 | 0.001144 | 0.000819 | 0.00084 | 0.000955 | 0.000923 | 0.000813 | 0.000801 | 0.000829 | 0.000835 | 0.000753 | 0.000749 |
| 9 | 0.000777 | 0.001513 | 0.000846 | 0.000868 | 0.000767 | 0.000742 | 0.000868 | 0.000849 | 0.000754 | 0.000734 | 0.000782 | 0.000831 | 0.000713 | 0.00072 |
| 10 | 0.000864 | 0.00153 | 0.000912 | 0.001107 | 0.0008 | 0.000803 | 0.000961 | 0.000958 | 0.000796 | 0.000782 | 0.000803 | 0.000806 | 0.000711 | 0.000725 |
| 11 | 0.00078 | 0.001549 | 0.000831 | 0.000959 | 0.000772 | 0.000753 | 0.000912 | 0.000892 | 0.000733 | 0.001015 | 0.000776 | 0.000802 | 0.000717 | 0.000751 |
| 12 | 0.000845 | 0.001561 | 0.000869 | 0.000939 | 0.000786 | 0.000763 | 0.000905 | 0.000849 | 0.000752 | 0.000755 | 0.000767 | 0.000806 | 0.000709 | 0.000739 |
| 13 | 0.000846 | 0.001344 | 0.000914 | 0.000987 | 0.000797 | 0.000826 | 0.000904 | 0.000886 | 0.000782 | 0.000796 | 0.000809 | 0.000849 | 0.000744 | 0.000756 |
| 14 | 0.000872 | 0.001416 | 0.000844 | 0.00089 | 0.000793 | 0.000807 | 0.00095 | 0.000944 | 0.000805 | 0.00079 | 0.000791 | 0.000791 | 0.000729 | 0.000748 |
| 15 | 0.001087 | 0.002077 | 0.001028 | 0.001216 | 0.000825 | 0.000774 | 0.000984 | 0.000902 | 0.000795 | 0.000786 | 0.000824 | 0.00082 | 0.000766 | 0.000765 |
| 16 | 0.000859 | 0.001518 | 0.000863 | 0.000876 | 0.000774 | 0.000781 | 0.000897 | 0.000845 | 0.000748 | 0.000748 | 0.000782 | 0.000806 | 0.000726 | 0.000748 |
| 17 | 0.000859 | 0.001557 | 0.000908 | 0.00093 | 0.000796 | 0.000791 | 0.000932 | 0.000879 | 0.000755 | 0.000756 | 0.000775 | 0.000808 | 0.000724 | 0.000744 |
| 18 | 0.0009 | 0.001521 | 0.000899 | 0.000878 | 0.000802 | 0.000803 | 0.000937 | 0.00084 | 0.000888 | 0.000778 | 0.000836 | 0.000778 | 0.000843 | 0.000726 |
| 19 | 0.000872 | 0.001657 | 0.001034 | 0.000988 | 0.000853 | 0.000849 | 0.001042 | 0.000896 | 0.000888 | 0.000876 | 0.000866 | 0.000849 | 0.000767 | 0.000799 |

CC, splenium of the corpus callosum; FNX, body of the fornix; SS, sagittal stratum; CGC, cingulum in the cingulate gyrus;

ST, stria terminalis; SLF, superior longitudinal fasciculus; UF, uncinate fasciculus; ALIC, anterior limb of internal capsule; R, right; L, left.

**Supplementary material 4** | Associations between mean FA/MD and Estimated IQ (Controlled for age and gender)

**FA MD**

| Variable | Coef | R-squared | p | Conf.Interval | t | Coef | R-squared | p | Conf.Interval | t |
| --- | --- | --- | --- | --- | --- | --- | --- | --- | --- | --- |
| **FNX** | **1.03×10^-3^** | **0.264** | **0.04** | **4.7×10^-5^~2.0×10^-3^** | **2.23** | **-4.85×10^-6^** | **0.332** | **0.04** | **-9.5×10^-6^~-1.98×10^-7^** | **-2.22** |
| **ST-R** | **9.89×10^-4^** | **0.514** | **0.001** | **4.5×10^-4^~ 1.5×10^-3^** | **3.89** | **-2.13×10^-6^** | **0.279** | **0.05** | **-4.3×10^-6^~2.3×10^-8^** | **-2.11** |
| **ST-L** | **1.02×10^-3^** | **0.617** | **0.000** | **5.5×10^-4^~ 1.5×10^-3^** | **4.66** | **-1.79×10^-6^** | **0.503** | **0.009** | **-3.1×10^-6^~ -5.2×10^-7^** | **-3.01** |
| **SS-R** | **7.51×10^-4^** | **0.521** | **0.001** | **3.5×10^-4^~ 1.1×10^-3^** | **4.03** | **-1.81×10^-6^** | **0.454** | **0.004** | **-3.0×10^-6^~ -6.7×10^-7^** | **-3.39** |
| **SS-L** | **6.2×10^-4^** | **0.365** | **0.011** | **1.7×10^-4^~ 1.1×10^-3^** | **2.92** | -2.08×10^-6^ | 0.295 | 0.065 | -4.3×10^-6^~ 1.5×10^-7^ | -1.99 |
| **UF-R** | **9.14×10^-4^** | **0.371** | **0.015** | **2.0×10^-4^~ 1.6×10^-3^** | **2.73** | **-8.07×10^-7^** | **0.479** | **0.002** | **-1.3×10^-6^~ -3.4×10^-7^** | **-3.65** |
| UF-L | 6.44×10^-4^ | 0.274 | 0.067 | -5.1×10^-5^~ 1.3×10^-3^ | 1.98 | -3.77×10^-7^ | 0.238 | 0.097 | -8.3×10^-7^~ 7.6×10^-8^ | -1.77 |
| CGC-R | 5.2×10^-4^ | 0.28 | 0.075 | -5.9×10^-5^~ 1.1×10^-3^ | 1.92 | **-6.27×10^-7^** | **0.531** | **0.002** | **-9.8×10^-7^~ -2.8×10^-7^** | **-3.81** |
| **CGC-L** | **6.2×10^-4^** | **0.31** | **0.047** | **10.0×10^-6^~ 1.2×10^-3^** | **2.17** | **-7.29×10^-7^** | **0.452** | **0.004** | **-1.2×10^-6^~ -2.8×10^-7^** | **-3.45** |
| **SLF-R** | **4.41×10^-4^** | **0.356** | **0.026** | **6.2×10^-5^~ 8.2×10^-4^** | **2.48** | **-1.09×10^-6^** | **0.659** | **0.000** | **-1.6×10^-6^~ -6.1×10^-7^** | **-4.82** |
| **SLF-L** | **4.01×10^-4^** | **0.451** | **0.014** | **9.5×10^-5^~ 7.1×10^-4^** | **2.79** | -6.54×10^-7^ | 0.161 | 0.230 | -1.8×10^-6^~ 4.6×10^-7^ | -1.25 |
| **CC** | **7.77×10^-4^** | **0.429** | **0.005** | **2.7×10^-4^~ 1.3×10^-3^** | **3.27** | **-1.59×10^-6^** | **0.415** | **0.007** | **-2.7×10^-6^~ -5.1×10^-7^** | **-3.13** |
| **ALIC-R** | **7.52×10^-4^** | **0.685** | **0.000** | **4.1×10^-4^~ 1.1×10^-3^** | **4.68** | **-7.20×10^-7^** | **0.527** | **0.005** | **-1.2×10^-6^~ -2.6×10^-7^** | **-3.33** |
| **ALIC-L** | **7.70×10^-4^** | **0.652** | **0.000** | **4.5×10^-4^~ 1.1×10^-3^** | **5.05** | **-7.98×10^-7^** | **0.501** | **0.002** | **-1.2×10^-6^~ -3.5×10^-7^** | **-3.81** |

Coef, coefficient; Conf.Interval, 95% confidence interval. FNX, body of the fornix; ST, stria terminalis; SS, sagittal stratum; UF, uncinate fasciculus; CGC, cingulum in the cingulate gyrus; SLF, superior longitudinal fasciculus; CC, splenium of the corpus callosum; ALIC, anterior limb of internal capsule; R, right; L, left.

**Supplementary materials 5** | Associations between FA/MD and Socialization score (Controlled for age and gender)

**FA MD**

| Variable | Coef | R-squared | p | Conf.Interval | t | Coef | R-squared | p | Conf.Interval | t |
| --- | --- | --- | --- | --- | --- | --- | --- | --- | --- | --- |
| **FNX** | **1.89×10^-3^** | **0.481** | **0.002** | **7.9×10^-4^~3.0×10^-3^** | **3.66** | **-8.72×10^-6^** | **0.511** | **0.003** | **-1.4×10^-5^~-3.4×10^-6^** | **-3.50** |
| **ST-R** | **1.57×10^-3^** | **0.718** | **0.000** | **1.0×10^-3^~ 2.1×10^-3^** | **6.08** | **-3.5×10^-6^** | **0.39** | **0.013** | **-6.1×10^-6^~-8.6×10^-7^** | **-2.83** |
| **ST-L** | **1.48×10^-3^** | **0.716** | **0.000** | **9.4×10^-4^~ 2.0×10^-3^** | **5.88** | **-2.79×10^-6^** | **0.612** | **0.001** | **-4.3×10^-6^~ -1.3×10^-6^** | **-3.98** |
| **SS-R** | **1.17×10^-3^** | **0.710** | **0.000** | **7.6×10^-4^~ 1.6×10^-3^** | **6.05** | **-2.7×10^-6^** | **0.555** | **0.001** | **-4.1×10^-6^~ -1.3×10^-6^** | **-4.18** |
| **SS-L** | **9.6×10^-4^** | **0.488** | **0.002** | **4.2×10^-4^~ 1.5×10^-3^** | **3.77** | **-3.34×10^-6^** | **0.379** | **0.022** | **-6.1×10^-6^~ -5.6×10^-7^** | **-2.56** |
| **UF-R** | **1.3×10^-3^** | **0.396** | **0.011** | **3.4×10^-4^~ 2.2×10^-3^** | **2.90** | **-1.2×10^-6^** | **0.593** | **0.000** | **-1.8×10^-6^~ -6.5×10^-7^** | **-4.62** |
| UF-L | 7.69×10^-4^ | 0.236 | 0.105 | -1.8×10^-4^~ 1.7×10^-3^ | 1.72 | -5.00×10^-7^ | 0.235 | 0.099 | -1.1×10^-6^~ 1.1×10^-7^ | -1.76 |
| CGC-R | 6.3×10^-4^ | 0.246 | 0.113 | -1.7×10^-4^~ 1.4×10^-3^ | 1.68 | **-7.53×10^-7^** | **0.444** | **0.007** | **-1.3×10^-6^~ -2.5×10^-7^** | **-3.16** |
| **CGC-L** | **9.06×10^-4^** | **0.348** | **0.028** | **1.1×10^-4^~ 1.7×10^-3^** | **2.44** | **-8.62×10^-7^** | **0.358** | **0.013** | **-1.5×10^-6^~ -2.1×10^-7^** | **-2.83** |
| **SLF-R** | **5.87×10^-4^** | **0.354** | **0.026** | **8.0×10^-5^~ 1.1×10^-3^** | **2.47** | **-1.34×10^-6^** | **0.579** | **0.001** | **-2.1×10^-6^~ -6.3×10^-7^** | **-4.00** |
| **SLF-L** | **5.49×10^-4^** | **0.466** | **0.011** | **1.5×10^-4^~ 9.5×10^-4^** | **2.91** | -1.15×10^-6^ | 0.227 | 0.11 | -2.6×10^-6^~ 2.7×10^-7^ | -1.72 |
| **CC** | **1.22×10^-3^** | **0.586** | **0.000** | **6.4×10^-4^~ 1.8×10^-3^** | **4.51** | **-2.32×10^-6^** | **0.489** | **0.002** | **-3.7×10^-6^~ -9.7×10^-7^** | **-3.66** |
| **ALIC-R** | **1.07×10^-3^** | **0.750** | **0.000** | **6.6×10^-4^~ 1.4×10^-3^** | **5.60** | **-8.99×10^-7^** | **0.483** | **0.009** | **-1.5×10^-6^~ -2.6×10^-7^** | **-2.98** |
| **ALIC-L** | **1.14×10^-3^** | **0.783** | **0.000** | **7.9×10^-4^~ 1.5×10^-3^** | **7.07** | **-9.33×10^-7^** | **0.388** | **0.009** | **-1.6×10^-6^~ -2.7×10^-7^** | **-3.01** |

Coef, coefficient; Conf.Interval, 95% confidence interval. FNX, body of the fornix; ST, stria terminalis; SS, sagittal stratum; UF, uncinate fasciculus; CGC, cingulum in the cingulate gyrus; SLF, superior longitudinal fasciculus; CC, splenium of the corpus callosum; ALIC, anterior limb of internal capsule; R, right; L, left.

**Supplementary materials 6** | Associations between FA/MD and Maladaptive behavior index (Controlled for age and gender)

**FA　　　　　　　　　　　　　　　　　　　　　　　　　　　　　　　　MD**

| Variable | Coef | R-squared | p | Conf.Interval | t | Coef | R-squared | p | Conf.Interval | t |
| --- | --- | --- | --- | --- | --- | --- | --- | --- | --- | --- |
| **FNX** | **-2.47×10^-2^** | **0.59** | **0.001** | **-3.8×10^-2^~ -1.2×10^-2^** | **-4.02** | **1.2×10^-4^** | **0.602** | **0.002** | **5.3×10^-5^~1.8×10^-4^** | **3.89** |
| **ST-R** | **-1.44×10^-2^** | **0.48** | **0.008** | **-2.4×10^-2^~ -4.5×10^-3^** | **-3.11** | **4.2×10^-5^** | **0.465** | **0.019** | **8.1×10^-6^~7.5×10^-5^** | **2.66** |
| **ST-L** | **-1.2×10^-2^** | **0.423** | **0.026** | **-2.2×10^-2^~ ^-^1.6×10^-3^** | **-2.48** | 1.14×10^-5^ | 0.332 | 0.374 | -1.5×10^-5^~ 3.8×10^-5^ | 0.92 |
| **SS-R** | **-9.08×10^-3^** | **0.279** | **0.041** | **-1.8×10^-2^~ 4.4×10^-4^** | **-2.25** | 1.7×10^-5^ | 0.361 | 0.112 | -4.5×10^-6^~ 3.9×10^-5^ | 1.70 |
| SS-L | -7.7×10^-3^ | 0.235 | 0.084 | -1.7×10^-2^~ 1.2×10^-3^ | -1.86 | 2.52×10^-5^ | 0.264 | 0.21 | -1.6×10^-5^~ 6.6×10^-5^ | 1.31 |
| UF-R | -1.2×10^-2^ | 0.189 | 0.105 | -2.6×10^-2^~ 2.8 ×10^-3^  ×10^-5^ | -1.73 | 5.9×10^-6^ | 0.307 | 0.202 | -3.5×10^-6^~ 1.5×10^-5^ | 1.34 |
| UF-L | -4.9×10^-3^ | 0.049 | 0.444 | -1.8×10^-2^~ 8.5×10^-3^ | -0.79 | 3.05×10^-6^ | 0.373 | 0.384 | -4.2×10^-6^~ 1.03×10^-5^ | 0.9 |
| CGC-R | -4.64×10^-3^ | 0.23 | 0.378 | -1.6×10^-2^~6.3×10^-3^ | -0.91 | 5.3×10^-6^-7.53×10^-7^ | 0.154 | 0.16 | -2.4×10^-6^~ 1.3×10^-5^ | 1.48 |
| CGC-L | -8.36×10^-3^ | 0.227 | 0.148 | -2.0×10^-2^~ 3.3×10^-3^ | -1.53 | 3.82×10^-6^ | 0.076 | 0.421 | -6.1×10^-6^~ 1.4×10^-5^ | 0.83 |
| SLF-R | -3.8×10^-4^ | 0.05 | 0.915 | -7.9×10^-3^~ 7.1×10^-3^ | -0.11 | 7.45×10^-6^ | 0.111 | 0.224 | -5.1×10^-6^~ 0.2×10^-4^ | 1.27 |
| SLF-L | -1.02×10^-3^ | 0.168 | 0.752 | -7.8×10^-3^~ 5.8×10^-3^ | -0.32 | 8.28×10^-6^ | 0.174 | 0.38 | -1.1×10^-5^~ 2.8×10^-5^ | 0.91 |
| CC | -1.02×10^-2^ | 0.244 | 0.055 | -0.02~ 0.24×10^-3^ | -2.1 | **2.75×10^-5^** | **0.41** | **0.01** | **7.8×10^-6^~ 4.7×10^-5^** | **3.00** |
| ALIC-R | -8.28×10^-3^ | 0.322 | 0.053 | -1.67×10^-2^~ 1.2×10^-4^ | -2.11 | 5.70×10^-6^ | 0.256 | 0.257 | -4.6×10^-6^~ 1.6×10^-5^ | 1.18 |
| ALIC-L | -6.10×10^-3^ | 0.278 | 0.144 | -1.45×10^-2^~ 2.3×10^-3^ | -1.55 | 5.79×10^-7^ | 0.139 | 0.237 | -4.3×10^-6^~ 1.6×10^-5^ | 1.23 |

Coef, coefficient; Conf.Interval, 95% confidence interval. FNX, body of the fornix; ST, stria terminalis; SS, sagittal stratum; UF, uncinate fasciculus; CGC, cingulum in the cingulate gyrus; SLF, superior longitudinal fasciculus; CC, splenium of the corpus callosum; ALIC, anterior limb of internal capsule; R, right; L, left.

| Variable | Coef | R-squared | p | Conf.Interval | t | Coef | R-squared | p | Conf.Interval | t |
| --- | --- | --- | --- | --- | --- | --- | --- | --- | --- | --- |
| **FNX** | **-1.91×10^-3^** | **0.445** | **0.032** | **-3.6×10^-3^~1.9×10^-4^** | **-2.40** | **1.03×10^-5^** | **0.507** | **0.017** | **2.2×10^-6^~1.9×10^-5^** | **2.75** |
| **ST-R** | **-1.3×10^-3^** | **0.63** | **0.011** | **-2.2×10^-3^~ ^-^3.6×10^-4^** | **-2.99** | 3.4×10^-6^ | 0.461 | 0.071 | -3.34×10^-7^~7.2×10^-6^ | 1.97 |
| **ST-L** | **-1.2×10^-3^** | **0.537** | **0.025** | **-2.3×10^-3^~ ^-^1.8×10^-4^** | **-2.53** | 2.01×10^-6^ | 0.42 | 0.134 | -7.1×10^-7^~ 4.7×10^-6^ | 1.60 |
| SS-R | -7.6×10^-4^ | 0.34 | 0.095 | -1.7×10^-3^~ 1.5×10^-4^ | -1.80 | 1.61×10^-6^ | 0.416 | 0.158 | -7.1×10^-7^~ 3.9×10^-6^ | 1.50 |
| SS-L | -7.93×10^-4^ | 0.463 | 0.056 | -1.6×10^-3^~ 2.2×10^-5^ | -2.10 | 2.7×10^-6^ | 0.236 | 0.231 | -1.9×10^-6^~ 7.3×10^-6^ | 1.26 |
| UF-R | -8.2×10^-4^ | 0.089 | 0.305 | -2.5×10^-3^~ 8.3×10^-4^ | -1.07 | 4.4×10^-7^ | 0.674 | 0.216 | -2.87×10^-7^~1.2×10^-6^ | 1.30 |
| UF-L | -9.6×10^-5^ | 0.016 | 0.893 | -1.6×10^-3^~ 1.4×10^-3^ | -0.14 | 2.50×10^-7^ | 0.262 | 0.526 | -5.8×10^-7^~ 1.08×10^-6^ | 0.65 |
| CGC-R | -3.92×10^-4^ | 0.411 | 0.445 | -1.47×10^-3^~6.83×10^-4^ | -0.79 | 7.72×10^-7^ | 0.293 | 0.053 | -1.2×10^-8^~1.6×10^-6^ | 2.13 |
| CGC-L | -7.8×10^-4^ | 0.443 | 0.150 | -1.9×10^-3^~ 3.2×10^-4^ | -1.53 | 7.69×10^-7^ | 0.250 | 0.122 | -2.3×10^-7^~ 1.8×10^-6^ | 1.66 |
| SLF-R | -1.9×10^-4^ | 0.182 | 0.61 | -9.5×10^-4^~ 5.8×10^-4^ | -0.52 | **8.83×10^-7^** | **0.522** | **0.046** | **1.6×10^-8^~ 1.8×10^-6^** | **2.20** |
| SLF-L | -3.7×10^-4^ | 0.313 | 0.273 | -1.1×10^-3^~ 3.2×10^-4^ | -1.14 | 1.34×10^-6^ | 0.230 | 0.19 | -7.7×10^-7^~ 3.4×10^-6^ | 1.37 |
| CC | -1.08×10^-3^ | 0.298 | 0.053 | -2.17×10^-3^~ 1.69×10^-5^ | -2.13 | **2.67×10^-6^** | **0.367** | **0.026** | **3.7×10^-7^~ 5.0×10^-6^** | **2.51** |
| **ALIC-R** | **-8.29×10^-4^** | **0.515** | **0.012** | **-1.44×10^-3^~ ^-^2.2×10^-4^** | **-2.93** | 6.06×10^-7^ | 0.156 | 0.174 | -3.1×10^-7^~ 1.5×10^-6^ | 1.44 |
| ALIC-L | -8.14×10^-4^ | 0.458 | 0.052 | -1.63×10^-3^~ 8.5×10^-6^ | -2.14 | 9.89×10^-7^ | 0.263 | 0.059 | -4.4×10^-8^~ 2.1×10^-6^ | 2.07 |

**Supplementary materials 7** | Associations between FA/MD and SRS-T score (Controlled for age and gender)

**FA MD**

Coef, coefficient; Conf.Interval, 95% confidence interval. FNX, body of the fornix; ST, stria terminalis; SS, sagittal stratum; UF, uncinate fasciculus; CGC, cingulum in the cingulate gyrus; SLF, superior longitudinal fasciculus; CC, splenium of the corpus callosum; ALIC, anterior limb of internal capsule; R, right; L, left.

| Variable | Coef | R-squared | p | Conf.Interval | t | Coef | R-squared | p | Conf.Interval | t |
| --- | --- | --- | --- | --- | --- | --- | --- | --- | --- | --- |
| FNX | -2.63×10^-2^ | 0.043 | 0.552 | -0.1~6.6×10^-2^ | -0.61 | 9.20×10^-5^ | 0.123 | 0.660 | -3.4×10^-4^~5.3×10^-4^ | 0.45 |
| ST-R | -5.12×10^-2^ | 0.220 | 0.071 | -0.1~5.0×10^-3^ | -1.94 | 4.8×10^-5^ | 0.081 | 0.615 | -1.5×10^-4^~2.5×10^-4^ | 0.51 |
| ST-L | **-5.7×10^-2^** | **0.323** | **0.03** | **-0.1~- 6.4×10^-3^** | **-2.40** | **1.68×10^-4^** | **0.596** | **0.002** | **7.4×10^-5^~ 2.6×10^-4^** | **3.82** |
| SS-R | **-4.5×10^-2^** | **0.280** | **0.03** | **-8.5×10^-2^~ -5.1×10^-3^** | **-2.40** | **1.38×10^-4^** | **0.395** | **0.009** | **3.9×10^-5^~ 2.4×10^-4^** | **2.98** |
| SS-L | **-4.83×10^-2^** | **0.330** | **0.016** | **-8.7×10^-2^~ -0.01** | **-2.70** | 1.4×10^-4^ | 0.239 | 0.130 | -4.7×10^-5^~ 3.3×10^-4^ | 1.60 |
| UF-R | -1.2×10^-2^ | 0.07 | 0.730 | -8.3×10^-2^~ 6.0×10^-2^ | -0.35 | **6.9×10^-5^** | **0.515** | **0.001** | **3.15×10^-5^~1.1×10^-4^** | **3.93** |
| UF-L | -5.6×10^-4^ | 0.085 | 0.985 | -6.5×10^-2^~ 6.3×10^-2^ | -0.02 | 1.98×10^-5^ | 0.144 | 0.301 | -2.0×10^-5^~ 5.9×10^-5^ | 1.07 |
| CGC-R | -3.7×10^-2^ | 0.231 | 0.135 | -8.6×10^-2^~1.3×10^-2^ | -1.58 | **4.03×10^-5^** | **0.355** | **0.022** | **6.6×10^-6^~7.4×10^-5^** | **2.55** |
| CGC-L | -3.9×10^-2^ | 0.219 | 0.137 | -9.3×10^-2^~ 1.4×10^-2^ | -1.57 | **4.51×10^-5^** | **0.264** | **0.040** | **2.3×10^-6^~ 8.8×10^-5^** | **2.25** |
| SLF-R | **-3.3×10^-2^** | **0.315** | **0.043** | **-6.5×10^-2^~ -1.2×10^-3^** | **-2.21** | **8.0×10^-5^** | **0.548** | **0.002** | **3.4×10^-5^~ 1.3×10^-4^** | **3.72** |
| SLF-L | **-3.0×10^-2^** | **0.401** | **0.028** | **-5.6×10^-2^~ - 3.7×10^-3^** | **-2.43** | 5.21×10^-5^ | 0.156 | 0.244 | -4.0×10^-5^~ 1.4×10^-4^ | 1.21 |
| CC | -4.77×10^-2^ | 0.250 | 0.050 | -9.5×10^-2^~ -8.6×10^-5^ | -2.14 | 5.33×10^-5^ | 0.096 | 0.320 | -5.7×10^-5^~ 1.6×10^-4^ | 1.03 |

**Supplementary materials 8** | Associations between FA/MD and refractory epilepsy (Controlled for age and gender).

**FA MD**

Coef, coefficient; Conf.Interval, 95% confidence interval. FNX, body of the fornix; ST, stria terminalis; SS, sagittal stratum; UF, uncinate fasciculus; CGC, cingulum in the cingulate gyrus; SLF, superior longitudinal fasciculus; CC, splenium of the corpus callosum; R, right; L, left.

**Supplementary materials 9** |Use of mTOR inhibitors and FA values in the left sagittal stratum (SS) in subjects with TSC

SS, sagittal stratum; mTOR-I (-), not using mTOR-Inhibitors; mTOR-I (+), using mTOR-Inhibitors

1= mTOR-Inhibitors for refractory epilepsy;　2= mTOR-Inhibitors for subependymal giant cell astrocytoma (SEGA)

1


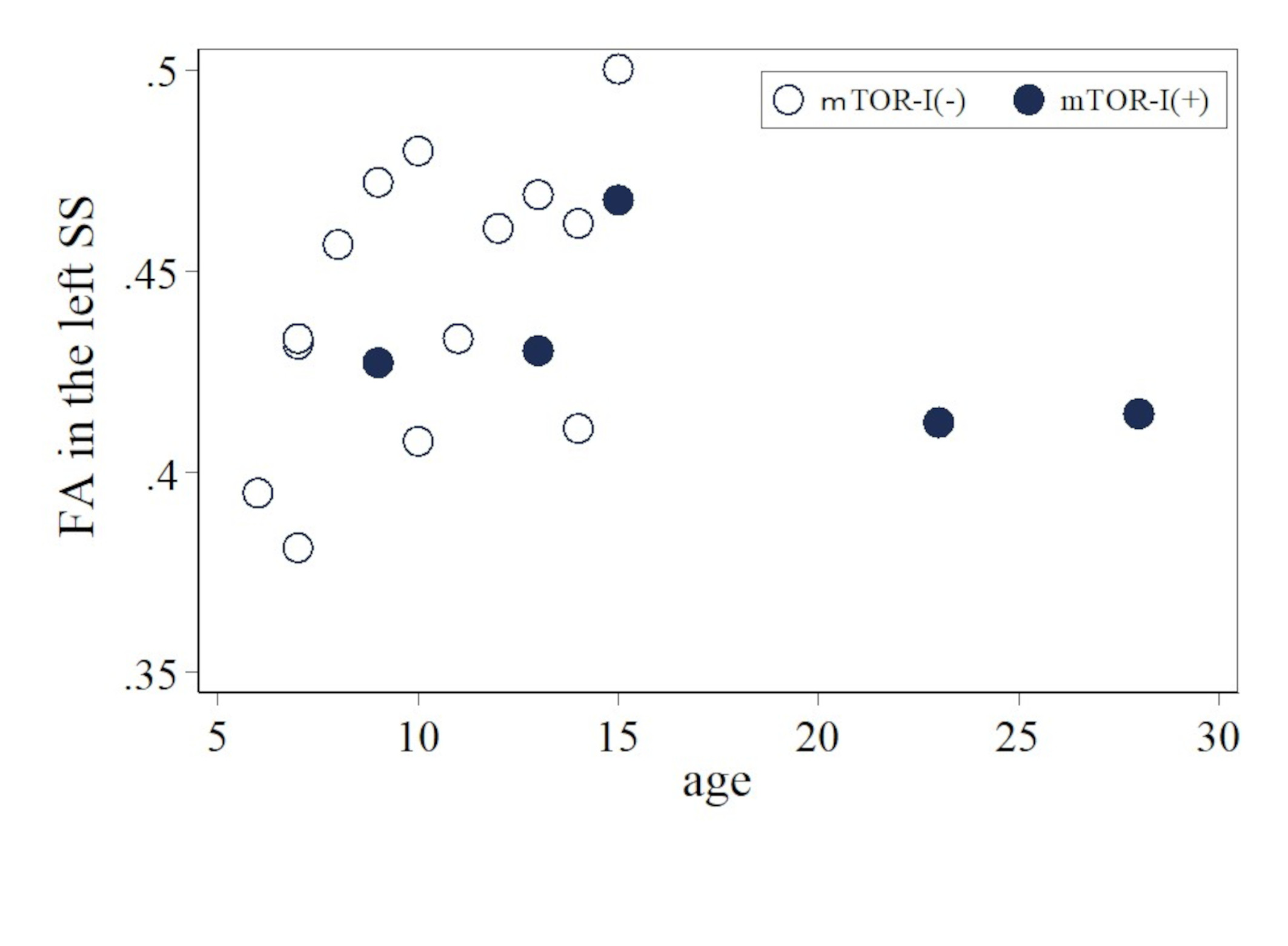


1

1

2

1

1
